# A Conserved HIV-1-Derived Peptide Presented by HLA-E Renders Infected T-cells Highly Susceptible to Attack by NKG2A/CD94-Bearing Natural Killer Cells

**DOI:** 10.1371/journal.ppat.1005421

**Published:** 2016-02-01

**Authors:** Zachary B. Davis, Andrew Cogswell, Hamish Scott, Amanda Mertsching, Julie Boucau, Daniel Wambua, Sylvie Le Gall, Vicente Planelles, Kerry S. Campbell, Edward Barker

**Affiliations:** 1 Department of Immunology/Microbiology, Rush University Medical Center, Chicago, Illinois, United States of America; 2 Division of Infection and Immunity and Cell Signaling and Cell Death, The Walter and Eliza Hall Institute of Medical Research, Parkville, Victoria, Australia; 3 Department of Medical Biology, The University of Melbourne, Parkville, Victoria, Australia; 4 Ragon Institute of MGH, MIT and Harvard, Cambridge, Massachusetts, United States of America; 5 Department of Pathology, University of Utah, Salt Lake City, Utah, United States of America; 6 Fox Chase Cancer Center, Institute for Cancer Research, Philadelphia, Pennsylvania, United States of America; Emory University, UNITED STATES

## Abstract

Major histocompatibility class I (MHC-I)-specific inhibitory receptors on natural killer (NK) cells (iNKRs) tolerize mature NK cell responses toward normal cells. NK cells generate cytolytic responses to virus-infected or malignant target cells with altered or decreased MHC-I surface expression due to the loss of tolerizing ligands. The NKG2A/CD94 iNKR suppresses NK cell responses through recognition of the non-classical MHC-I, HLA-E. We used HIV-infected primary T-cells as targets in an *in vitro* cytolytic assay with autologous NK cells from healthy donors. In these experiments, primary NKG2A/CD94^+^ NK cells surprisingly generated the most efficient responses toward HIV-infected T-cells, despite high HLA-E expression on the infected targets. Since certain MHC-I-presented peptides can alter recognition by iNKRs, we hypothesized that HIV-1-derived peptides presented by HLA-E on infected cells may block engagement with NKG2A/CD94, thereby engendering susceptibility to NKG2A/CD94^+^ NK cells. We demonstrate that HLA-E is capable of presenting a highly conserved peptide from HIV-1 capsid (AISPRTLNA) that is not recognized by NKG2A/CD94. We further confirmed that HLA-C expressed on HIV-infected cells restricts attack by KIR2DL^+^ CD56^dim^ NK cells, in contrast to the efficient responses by CD56^bright^ NK cells, which express predominantly NKG2A/CD94 and lack KIR2DLs. These findings are important since the use of NK cells was recently proposed to treat latently HIV-1-infected patients in combination with latency reversing agents. Our results provide a mechanistic basis to guide these future clinical studies, suggesting that *ex vivo*-expanded NKG2A/CD94^+^ KIR2DL^-^ NK cells may be uniquely beneficial.

## Author Summary

A critical mechanism by which the immune system controls viruses is through destruction of infected cells. One way to avoid cytotoxic T-lymphocytes (CTL) is by down modulating ligands that present virus peptides, the major histocompatibility complex class I molecules (MHC-I). However, MHC-I negatively regulates natural killer (NK) cells. It was thought that one way that HIV avoids both CTLs and NK cells is by leaving ligands (HLA-C and–E) to NK cell inhibitory receptors (iNKRs) while down modulating HLA-A and -B. However, NK cell expression of specific iNKRs is variegated and as such only some, but not all, NK cells are affected. We demonstrate here that NK cells expressing HLA-E specific iNKRs (NKG2A/CD94) destroy HIV-infected cells despite expression of HLA-E. Peptides presented by HLA-E are critical for binding to iNKRs on NK cells. We show in this study that an HIV capsid peptide presented by HLA-E is unable to interact with NKG2A/CD94, and as such the NK cell expressing the HLA-E specific iNKR will lyse the infected cell. This is important given that viremia in HIV-infected patients is associated with an increase in circulating NK cells expressing HLA-C iNKRs but very few NK cells expressing HLA-E iNKRs.

## Introduction

It was realized even as early as the mid-1980s that HIV-1-infected cells are refractory to Natural Killer (NK) cell-induced lysis [1,2]. About fifteen years later it was revealed that HIV-1 selectively down-modulates HLA-A and -B to avoid cytotoxic T-lymphocyte (CTL) while leaving HLA-C and -E on the infected cell surface to counter destruction by NK cells [3].

Several studies implicate HLA-C in preventing NK cell lysis of HIV-infected cells. In addition to retention of HLA-C on the surface of HIV-infected cells, there is an increased frequency of NK cells expressing the HLA-C-specific iNKR, KIR2DL1, -2, or -3, in viremic subjects compared to aviremic subjects [4]. While retained expression of HLA-C diminishes NK cell responses to HIV-infected cells, HIV appears to preserve specific peptides that are presented by HLA-C in order to enhance NK cell inhibition [5]. Although, the ability of a specific HIV peptide to both stabilize HLA-C surface expression and trigger KIR2DLs is very limited and self-peptides on HIV-infected cells also inhibit KIR2DL-expressing NK cells [6].

In contrast to HLA-C, the role that HIV-specific peptides presented by HLA-E have in diminishing NK cells expressing HLA-E specific receptors (*i*.*e*., NKG2A/CD94) has not been fully evaluated. Peptides engage NKG2A/CD94 and are presented by HLA-E are limited to highly conserved peptide sequences, typically the leader peptide of MHC-I molecules [7]. In order for HLA-E to regulate NK cells, the MHC-I leader peptide must not only possess an optimized sequence for tight and deep binding into the HLA-E groove [8], but also must possess residues that interact with specific residues on NKG2A and CD94 on the NK cell [7,9,10]. Other peptides, which mimic the sequences of the leader peptide of MHC-I may also be presented by HLA-E and engage NKG2A/CD94 [11,12]. In some instances these alternative HLA-E-presented peptides are unlikely to interact with NKG2A/CD94 on NK cells [13].

Despite HLA-E expression on HIV-infected cells, the proportion of NK cells expressing HLA-E specific receptors (*i*.*e*., NKG2A/CD94) is significantly decreased in viremic subjects compared to NK cells from aviremic individuals [14,15,16]. Thus, we hypothesized that NK cells expressing NKG2A/CD94 may in fact be capable of lysing HIV-infected cells despite the presence of HLA-E on the infected cell surface. To test this hypothesis, we did a comprehensive analysis of the roles of HLA-E specific iNKRs in tolerizing NK cell-mediated cytolytic responses to autologous HIV-infected target cells and whether an HIV peptide reported to be presented by HLA-E on infected cells [17] is capable of engaging NKG2A/CD94 on NK cells.

## Results

### HLA-E on HIV-infected cells does not suppress the cytolytic response of NKG2A/CD94^+^ NK cells

**Fig 1:**
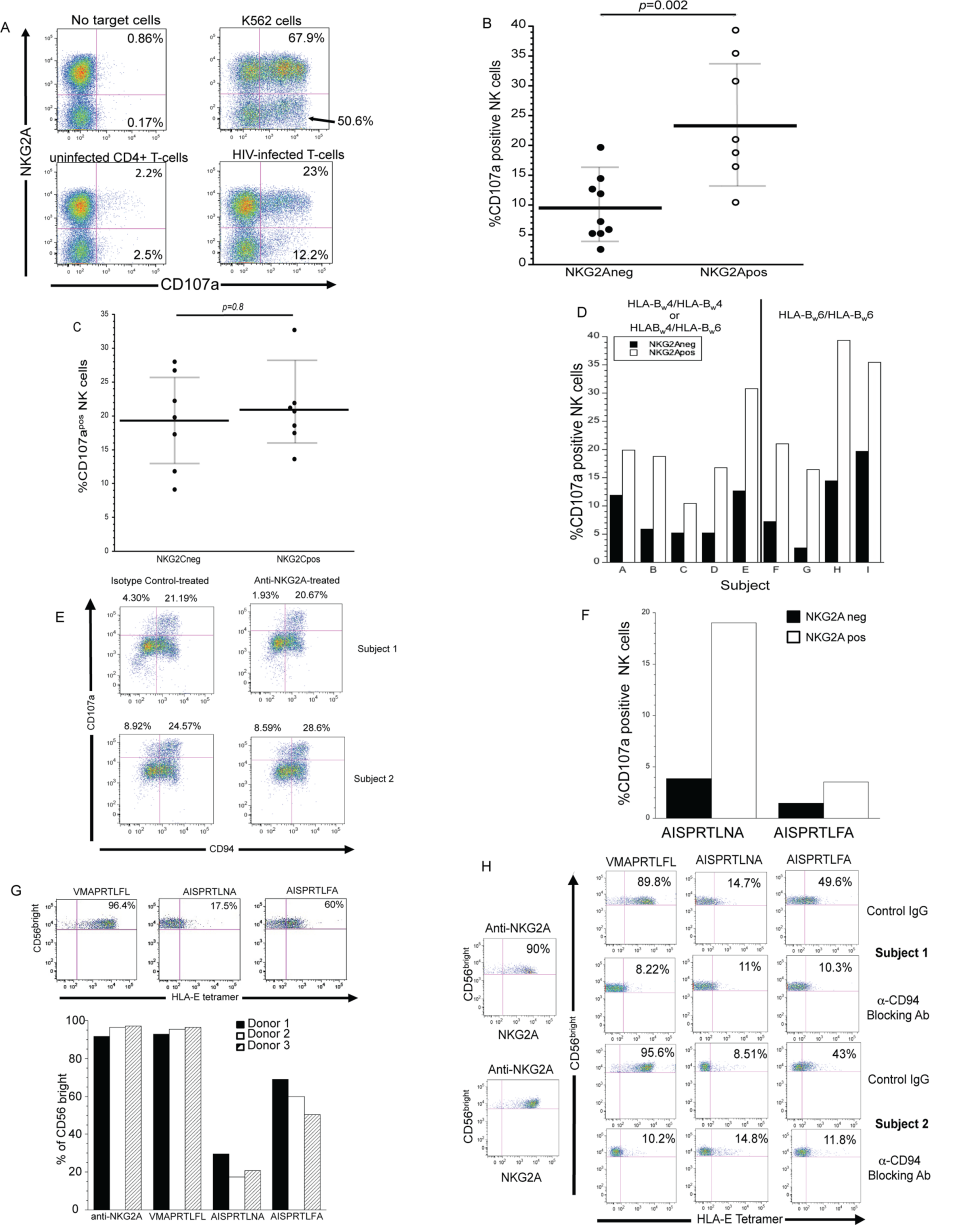
NK cells possessing NKG2A/CD94 degranulate in response to autologous HIV-infected T-cells despite HLA-E surface expression. (A) An example of the ability of NK cells possessing and lacking NKG2A/CD94 to degranulate following exposure to HIV-infected T-cells (from data presented in Fig 1B). As target cell controls, NK cells were exposed to K562 cells or uninfected CD4^+^ T-cells. NK cells ability to degranulate in the absence of target cells are also shown. Bold numbers are percent of NKG2A/CD94^+^ (upper quadrants) and NKG2A/CD94^-^ (lower quadrants) NK cells that express CD107a on their surface after 4-hour culture with or without target cells. (B) Ability of NK cells lacking (black dots) and expressing (white dots) NKG2A/CD94 from nine different donors to degranulate following exposure to HIV-infected cells. Statistical significance (p≤0.05) was determined using the Wilcoxon-ranked sum test. Black Bars represent the mean of the CD107a responses of each NK cell subpopulation of the different donors. Gray error bars represent the standard deviation of the mean. (C) NK cells, from seven different donors, which possess the HLA-E specific activation receptor NKG2C/CD94, degranulate to the same degree as NKG2C/CD94^-^ NK cells when exposed to autologous HIV-infected T-cells. Statistical significance (p≤0.05) was determined using the Wilcoxon-ranked sum test. Bars represent the mean of the CD107a responses of each NK cell subpopulation of the different donors. Gray error bars represent the standard deviation of the mean. (D) Expression of CD107a on NKG2A/CD94^-^ NK cells (black bars) and NKG2A/CD94^+^ NK cells (white bars) from five HLA-Bw4^+^ (subjects A-E), or four HLA-Bw6^+^/HLA-Bw6^+^ (subjects F-I) donors in response to HIV-infected T-cells. (E) Impact of anti-NKG2A blocking antibodies on degranulation of CD94+ NK cells when exposed to HIV-infected T-cells in the presence of Fab fragments of anti-CD16 blocking antibody. As a control, NK cells were treated with Fab fragments of anti-CD16 antibodies alone (absence of HIV-infected T-cells) and an antibody of similar isotype as anti-NKG2A (isotype control). Numbers above quadrants one and two designate the response (CD107a expression) of the CD94^-^ and CD94^+^ NK cells. (F) Effect of a point mutation in HIV-1 Gag on the ability of NKG2A- NK cells (black bars) and NKG2A^+^ (white bars) NK cells to degranulate when exposed to T-cells infected with HIV-1 with wild-type Gag (AISPRTLNA) or HIV-1 with an N to F mutation in position 153 of Gag (AISPRTLFA). This is an example of three experiments. (G) Purified NK cells stained with fluorochrome-conjugated HLA-E tetramer containing the leader peptide of HLA-G (VMAPRTLFL), wild type HIV-1 p24 capsid peptide (AISPRTLNA) or HIV-1 p24 capsid peptide with an asparagine to phenylalanine substitution (AISPRTLFA). As a control, the same purified NK cells were stained with antibody to NKG2A. Staining control consisted of NK cells stained with fluorochrome-conjugated IgG1. Dot Plots are examples of the data shown in the bottom panels. (H) Ability of HLA-E tetramer with peptide containing an N to F mutation in position 153 of Gag (AISPRTLFA) to interact with CD56^bright^ NK cells when blocking antibodies to NKG2A were present. As controls CD56^bright^ NK cells were stained with HLA-E tetramer with peptide containing the leader peptide of HLA-G (VMAPRTLFL) or wild type HIV-1 p24 capsid peptide (AISPRTLNA) in the presence and absence of blocking antibodies to NKG2A.

Surface expression of HLA-E is not modulated by HIV-1 (**S1A Fig**) on infected T-cells and is consequently expected to inhibit the cell-lysis-inducing activity of NK cells bearing NKG2A/CD94 receptors [3,18]. To test this expectation, purified primary HIV-infected T-cells (**S1B Fig**) were used as targets to stimulate degranulation by autologous NK cells. In contrast to the expected outcome, degranulation by NKG2A^+^ NK cells was significantly more robust (>2-fold; *p* = 0.002) than that by NK cells lacking the HLA-E-specific inhibitory receptor (**Fig 1A and 1B**).

Because NKG2A is disulfide-linked to CD94 [19], we determined the impact of CD94 expression on NK cell degranulation. Both CD94^+^ NK cells and NKG2A^+^ NK cells degranulated to a similar extent, which was significantly greater than NK cells lacking CD94 or NKG2A (**S1C Fig**). Given that the HLA-E specific activation receptor NKG2C is also disulfide-linked to CD94 [19], we determined whether enhanced responsiveness of CD94^+^ NK cells results from coupling to NKG2C. We found a relatively low frequency of NKG2C expressing NK cells in the peripheral blood of our donors (~5%). **S1D Fig** shows an example of NKG2C^+^ NK cells in the peripheral blood from one of our seven donors (the frequency of which was approximately 9%). Despite the low frequency of NKG2C/CD94 bearing NK cells in the subjects tested, the degranulation of NK cells from seven subjects in response to autologous HIV-infected cells was independent of NKG2C/CD94 expression (**Fig 1C**). We did not exclude NKG2A and NKG2C co-expressing NK cells from our analysis. It should be noted that a higher frequency of NKG2C/CD94+ NK cells possess KIR2DLs in comparison to NK cells that lack NKG2C (**S1E Fig**).

Another possible explanation for why NKG2A/CD94^+^ NK cells respond better to HIV-infected cells than NKG2A/CD94^-^ NK cells could be the presence of a higher frequency of KIR3DL1^+^ NK cells within the NKG2A/CD94-bearing NK cell subset. Studies point to a greater responsiveness of KIR3DL1^+^ NK cells to HIV-infected cells, due to the loss of HLA-Bw4 ligand [20]. However, we did not find any differences in the ability of KIR3DL1^+^ NK cells to degranulate in response to autologous HIV-infected T-cells regardless of whether they were from donors expressing HLA-Bw4 or from donors that were homozygous for HLA-Bw6 (**S1F Fig**). Only when we excluded both KIR2DL and NKG2A expressing NK cells from the analysis did we noted an increase in the ability of KIR3DL1^+^ NK cells from HLA-Bw4 donors to degranulate compared with KIR3DL1^-^ NK cells in response to infected cells, as expected (**S1G Fig**). The HLA-Bw4 status of the donor did not influence the capacity of NKG2A/CD94^+^ NK cells to degranulate in response to autologous HIV-infected T-cells (**Fig 1D**).

To determine whether NKG2A/CD94^+^ NK cells respond to HIV-infected cells despite the presence of HLA-E, we set out to determine if HLA-E on infected T-cells was triggering inhibition of NK cell activity. Blocking the interaction between NKG2A/CD94 on primary NK cells and its ligand HLA-E did not impact NK cell degranulation of CD94^+^ NK cells (**Fig 1E**). In contrast, the same antibody potentiated degranulation of NKG2A/CD94^+^ NK cells by at least 2-fold in response to a B-cell line expressing HLA-E (**S1H and S1I Fig**). This increased NK cell responsiveness to HLA-E expressing B-cell lines in the presence of NKG2A-blocking Ab was not due to the destruction of NK cells through antibody-dependent cell-mediated cytotoxicity (ADCC)-induced fratricide because FcγRIIIa was pre-blocked using the anti-CD16 Fab’ fragment (**S1I Fig**). In contrast, the anti-CD16 Fab’ fragment prevents NK cells from mediating ADCC against a Rituxan-treated B-cell line (**S1J and S1K Fig**). We also exposed our NK cells to anti-CD16 Fab’ fragment prior to exposure to NKG2A blocking antibody and HIV-infected T-cells (**Fig 1E and S1L Fig).** Based on our blocking studies, we posit that HLA-E on HIV-infected cells is incapable of inhibiting NK cells.

Based on our finding that NKG2A/CD94^+^ NK cells respond more robustly to HIV-infected T-cells than do NK cells lacking this HLA-E-specific iNKR, we postulated that HLA-E on HIV-infected cells is not recognized by NKG2A/CD94. Several studies support the notion that NKG2A/CD94-bearing NK cells appear to be licensed/educated during development to lyse targets lacking HLA-E [21,22,23,24,25]. Therefore NK cells possessing NKG2A/CD94 should have an increased response to target cells incapable of triggering this iNKR compared to NK cells lacking this HLA-E-specific receptor. Consistent with this hypothesis, NKG2A/CD94^+^ NK cells degranulated more extensively than did NK cells lacking NKG2A (*p* = 0.016) when exposed to K562 cells lacking surface HLA-E (**S1M Fig**).

Given that peptides within the HLA-E binding groove are involved in interactions with NKG2A/CD94 [7,26], we hypothesized that NKG2A/CD94 does not effectively engage HLA-E due to the presentation of an HIV-1-derived peptide. Previous studies [17] indicate that a highly conserved HIV-1 Gag peptide (AISPRTLNA or AA9) (**S2A Fig**) allows for HLA-E surface expression. Residues in position 2, 3, 5 and 6 of the peptide interact with HLA-E while residues in position 5 and 6 interact with NKG2A and a residue in position 8 interacts with CD94 [7,26]. The residue at position eight of the canonical MHC-I leader peptides loaded into the binding groove of HLA-E is typically a hydrophobic residue [7,26], whereas HIV-1 Gag peptide contains an asparagine at position eight.

MHC-bound HIV peptides are produced during the intracellular degradation of HIV virus or proteins inside cells [27]. The first step of protein degradation occurs in the cytosol and generates optimal epitopes and extended versions of epitopes. We have developed mass spectrometry-based degradation assays in cytosol of primary cells to follow the degradation of proteins into precursors and epitopes [28]. The assay allows us to measure the kinetics and amount of epitopes produced and is consistent with endogenous processing and presentation by target cells to T cells [28,29]. We assessed whether AA9 could be produced during the degradation of longer precursors (**S2 Fig**). Peptides encompassing AA9, a 12-mer (2-AA9-1) and a 35-mer (p24-10-35m) were degraded for 2 hours in the cytosol of CD3/28 activated CD4 T cells (**S2B Fig**). The degradation peptides were separated and identified by LC-MS/MS where each peptide is characterized by its mass, charges and peak intensity as illustrated in **S2C Fig** for 2-AA9-1. The degradation products included the original peptide, AA9, N-extended version of AA9 (1-, 2-AA9 for 2-AA9-1 and up to 3-AA9 for longer precursors), which could be further trimmed by endoplasmic reticulum-resident aminopeptidase into AA9, and antitopes (peptides that do not contain the full AA9 sequence). We detected both AA9 and N-extended precursors of AA9 during the degradation of 2-AA9-1 and p24-10-35m in cytosol from four different healthy donors (**S2D Fig**). We quantified the relative amount of AA9 and precursors by measuring the contribution of each peptide to the total intensity of degradation products. AA9 represented 0.02–0.1% while N-extended AA9 represented up to 0.13–1.7% of the total amount of peptides. Comparing AA9 production to that of neighboring T cell epitopes HLA-B57-restricted KF11 (KAFSPEVIPMF), which is processed from p24-10-35m in enough quantity to activate epitope-specific CTL [30,31], AA9 and precursors were produced in similar or higher quantities, as compared to precursors. These results show that AA9 and its N-extended precursors (which could be further trimmed in the ER) are made in the cytosol of CD3/28 activated CD4 T cells and could be presented by these cells.

Given that the amide residue at position eight of the AA9 is unlikely to interact with CD94 we wanted to determine whether mutating the corresponding residue at position 153 within HIV-1 Gag from an asparagine residue to a phenylalanine residue would allow HLA-E on the infected cells to interact with CD94 and prevent NK cell cytolytic response. Because N153 is highly conserved and is critical for HIV capsid structure [32], we generated a virus in which we provided HIV-1 proteins in *trans*, using a packing vector [33]. After infection we purified the infected cells and mixed them with autologous NK cells. We found a six-fold greater frequency of NKG2A/CD94^+^ NK cells that degranulated when exposed to purified wild-type virus-infected T-cells compared with the percent of NK cells that degranulated in response to purified mutant virus-infected T-cells (**Fig 1F**).

To further test the capacity of the AA9 Gag-derived peptide bound to HLA-E to interact with CD94, we determined the extent to which HLA-E tetramers loaded with AISPRTL**N**A or AISPRTL**F**A are capable of interacting with NK cells. As a positive control, we stained purified NK cells with HLA-E tetramers loaded with the canonical leader peptide of HLA-G (VMAPRTLFL). In another control, we stained purified NK cells with anti-NKG2A antibodies. Because CD56^dim^ NK cells show variable expression of NKG2A/CD94 among donors, and because CD56^bright^ NK cells are predominantly NKG2A/CD94^+^ in all donors tested, we evaluated the extent to which the tetramers bind to CD56^bright^ NK cells. While on average 62% of CD56^bright^ NK cells stained with HLA-E tetramers containing the mutant AISPRTL**F**A peptide, only 19% of CD56^bright^ NK cells stained with HLA-E tetramers containing wild type AISPRTL**N**A (**Fig 1G**). Furthermore, as shown in **Fig 1H** we found that HLA-E tetramers containing the mutated HIV AISPRTL**F**A peptide no longer interacts with CD56^bright^ NK cells when CD94 was blocked.

Together, our findings indicate NKG2A/CD94 on NK cells is unable to interact with HLA-E on HIV-infected T-cells most likely because HLA-E presents a highly conserved HIV peptide that does not bind to CD94. As a result, by incapacitating this tolerizing ligand, NKG2A^+^ NK cells acquire improved capacity to degranulate in response to HIV-infected T-cells.

### HLA-C on HIV-infected cells suppresses responses by KIR2DL^+^ NK cells

**Fig 2:**
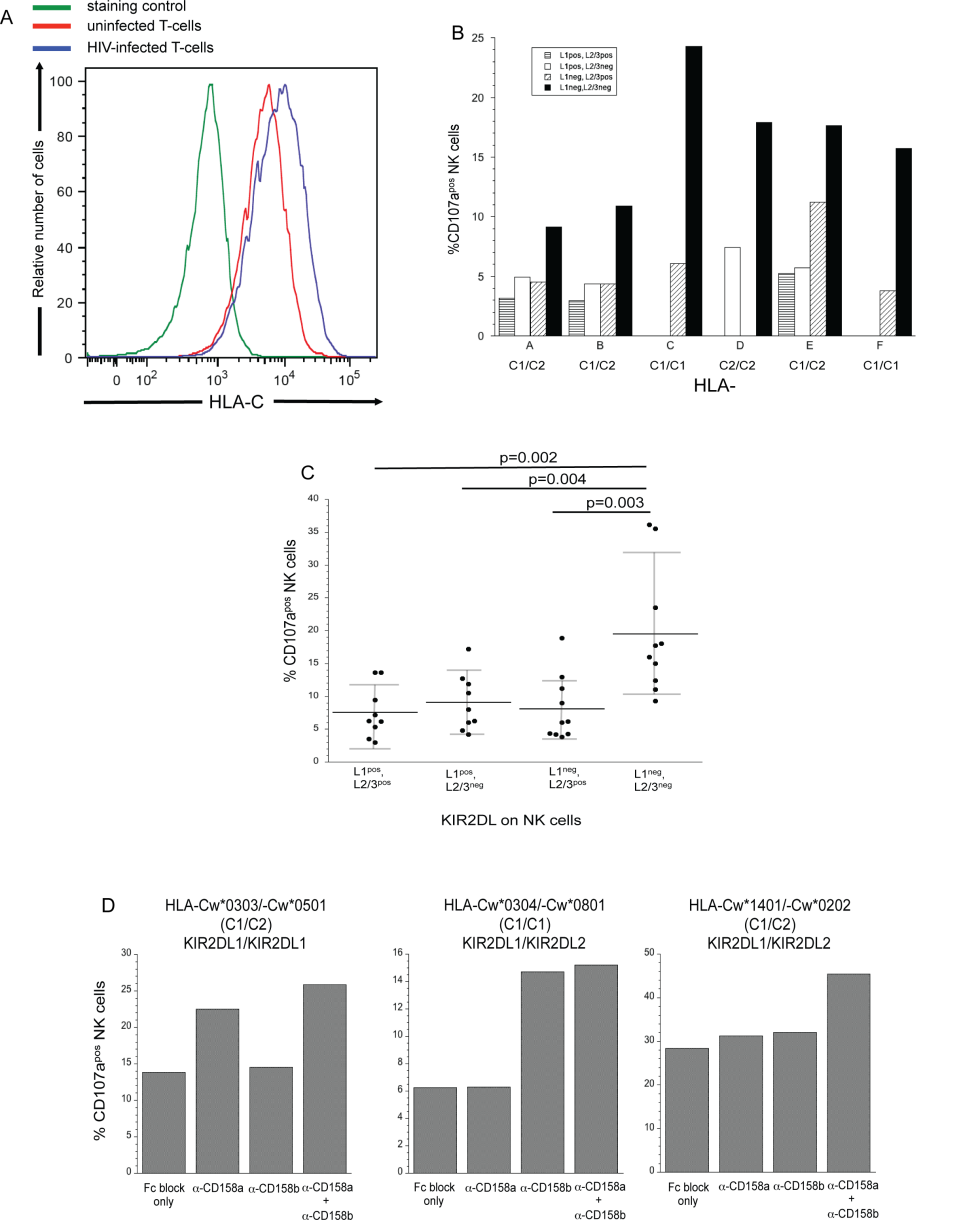
HLA-C on HIV-infected T-cells prevents KIR2DL expressing NK cells from degranulating. (A) Expression of HLA-C on HIV-infected T-cells [HIV-1 p24 antigen positive] (blue line) and uninfected (red line) CD4^+^ T-cells, as determined by soluble KIR2DL-IgG fusion protein staining and staining control (secondary antibody alone–green line). This is an example of three separate experiments. (B) Degranulation of NK cells expressing or lacking KIR2DL1 and/or KIR2DL2/3 from six donors with different HLA-C genotypes when exposed to HIV-infected T-cells. In general, KIR2DL1 recognizes group 2 HLA-C alleles (C2), while KIR2DL2/3 recognize group 1 HLA-C (C1). Degranulation responses from donors lacking specific KIR2DL alleles are not shown. (C) Ability of NK cells lacking or expressing the various HLA-C specific inhibitory receptors to degranulate when exposed to HIV-infected T-cells. Each point represents a specific donor; bars represent the mean response of a given population of NK cells. Gray error bars represent the standard deviation of the mean. Degranulation in response to exposure to HIV-infected cells was significantly different (p<0.05; Wilcoxon ranked sum test) between NK cells lacking all KIR2DL inhibitory receptors and NK cells possessing the indicated KIR2DLs. (D) Contributions of KIR2DL1 and/or KIR2DL2/3 on the ability of NK cells to degranulate when exposed to HIV-infected cells were determined by pretreating NK cells with blocking antibodies against KIR2DL1 (anti-CD158a) and/or KIR2DL2/3 (anti-CD158b). The HLA-C allele and KIR2DLs expressed by each donor are given above each figure. KIR2DL1 recognizes HLA-C with a lysine at position 80 (C2) while KIR2DL2/3 predominantly recognizes HLA-C molecules with an asparagine at position 80 (C1) but also may recognize HLA-C2. Fab’ fragments of CD16 blocking antibodies (Fc block) were added to all samples. Each plot represents a different subject in which the frequency of 2 X 10^4^ NK cells expressing CD107a was determined after 4-hour incubation with purified autologous HIV-infected T-cells in the absence or presence of anti-KIR2DL blocking antibodies.

Besides HLA-E, HLA-C remains on the surface of HIV-infected cells (**Fig 2B)**. Therefore, we hypothesized that in the context of HIV-infection, HLA-C will act alone in controlling NK cells. To test this hypothesis, we wanted to determine if NK cell subsets that differentially express KIR2DL1, -2 and -3 (**S3A Fig**) are inhibited by their HLA-C ligands on HIV-infected cells. Initially, we compared the capacities of NK cells lacking KIR2DLs with NK cells expressing KIR2DLs to degranulate in response to HIV-infected cells. Since KIR genes are differentially inherited in humans [34], not all donors tested expressed every KIR2DL. Hence, results from donors lacking genes for either KIR2DL1 or KIR2DL2/3 are not included in **Fig 2B and 2C**. NK cells lacking HLA-C-specific KIRs, on average, degranulated at 2-3-fold higher levels in response to HIV-infected T cells compared to NK cells possessing one or more KIR2DLs (**Fig 2B and 2C**). This suppressed responsiveness of KIR2DL^+^ NK cells corresponded with the HLA-C genotype of the donor (**Fig 2B**). We observed similar results regardless of the strain of virus used to infect the target cells (**S3B Fig**). We also noted that KIR2DL^+^ NK cells obtained from aviremic HIV-infected patients, like NK cells from uninfected subjects, had a diminished ability to degranulate in response to HIV-infected cells (*i*.*e*., CD4+ T-cells from the HIV-infected patients were infected with HIV-1 *in vitro* and infected cells purified 3–4 days later as described in Materials and Methods section). In contrast, the patients’ NKG2A/CD94^+^ NK cells had an increased ability relative to NKG2A/CD94^-^ NK cells to degranulate in response to HIV-infected cells (**S3C Fig**).

NK cells expressing or lacking KIR2DLs degranulate similarly in response to K562 cells, indicating that NK cells possessing KIR2DLs do not have a reduced capacity to degranulate against target cells lacking HLA-C (**S3D Fig**). Therefore, we hypothesized that HLA-C on infected cells decreases NK cell degranulation by engaging KIR2DLs. To test this hypothesis we treated NK cells with anti-KIR2DL1 and/or anti-KIR2DL2/3 blocking antibodies in the presence of anti-CD16 Fab’ fragments prior to exposure to HIV-infected T-cells (**Fig 2D**). Blocking of KIR2DLs on NK cells was specific, because NK cells degranulated at higher rates only when the corresponding KIR2DL was prevented from interacting with available cognate HLA-C ligand (**Fig 2D**). KIR2DL blocking antibodies did not trigger KIR2DS activation receptors, since NK cell degranulation did not occur when the blocking Abs were cross-linked by FcR on P815 cells (**S3E Fig**). In conclusion, our data demonstrate that NK cells expressing iNKRs for HLA-C (KIR2DL1/2/3) are inhibited from degranulating in response to autologous HIV-infected T-cells, indicating that retained HLA-C expression plays a prominent role in protecting HIV-1 from NK cell lytic responses.

### CD56^bright^ NK cells predominantly lyse autologous HIV-infected T-cells

**Fig 3:**
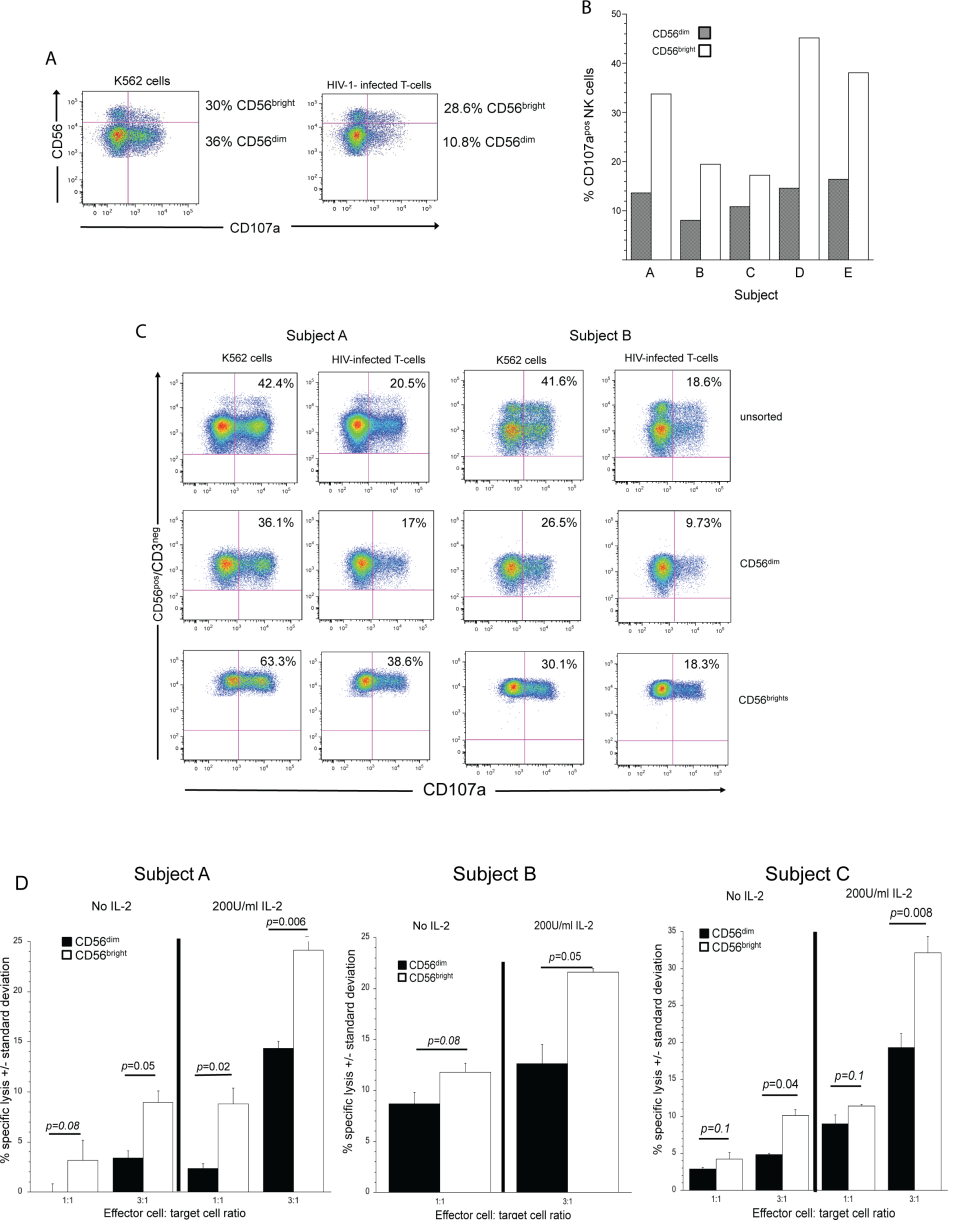
CD56^bright^ NK cells lyse autologous HIV-infected T-cells to a greater extent than CD56^dim^ NK cells. **(A)** An example of an experiment (from data presented in Fig 3B) to determine if CD107a is expressed on NK cell subsets expressing relatively high (CD56^bright^) or low (CD56^dim^) levels of CD56 when exposed to K562 cells or HIV-infected T-cells. Bold numbers to the right of the figures are percent of CD56^bright^ (upper quadrants) and CD56^dim^ (lower quadrants) NK cells that express CD107a on their surface after 4-hour exposure to target cells. (**B)** Percent of CD107a positive CD56^dim^ (black bars) and CD56^bright^ (white bars) NK cells from five subjects after exposure to autologous HIV-infected cells. (**C)** Ability of unsorted NK cells, sorted CD56^dim^ NK cells and sorted CD56^bright^ NK cells from two different donors (subjects A and B) to degranulate in response to K562 cells or HIV-infected T-cells. The HLA genotype for subject A was A*0201/A*1101 B*0702 (HLA-Bw6) /B*5601 (HLA-Bw6) and Cw*0102 (C1)/Cw*0401(C2). The HLA genotype for subject B was A*2901/A*1101 B*4403 (HLA-Bw4)/B*5101 (HLA-Bw4) and Cw*1502 (C2)/Cw*1601 (C1). Numbers in each plot represent the percent of CD107a^+^ NK cells of unfractionated, sorted CD56^dim^ or sorted CD56^bright^ NK cells after exposure to target cells. (**D)** Percent specific lysis of HIV-1 infected T-cells by sorted CD56^dim^ (black bars) and sorted CD56^bright^ (white bars) NK cells from three donors cultured overnight without or with 200 U/ml of IL-2. Error bars represent standard deviations of percent specific lysis of three replicates. The HLA genotype for the first subject was A*0201/A*1101, B*0702 (HLA-Bw6) /B*5601 (HLA-Bw6) and Cw*0102 (C1) /Cw*0401 (C2). The HLA genotype for the second subject was A*0101/A*2901, B*0801 (HAL-Bw6)/B*4403 (HLA-Bw4) and Cw*0701 (C1)/Cw*1601(C1). The HLA genotype for the third subject was A*1101/A*3101, B*5101 (HLA-Bw4)/B*4001 (HLA-Bw6) and Cw*0304 (C1)/Cw*1402 (C1). Numbers over bars between columns indicate *p*-values based on Mann-Whitney U-tests. *p*≤0.05 was considered statistically significant.

Taken together, our data demonstrate that the vast majority of NK cells lacking KIR2DLs and possessing NKG2A/CD94 have a greater capacity to degranulate than NK cells lacking NKG2A/CD94 and possessing KIR2DLs. Less then five percent of the peripheral blood CD56^bright^ NK cell subset expresses KIRs. In contrast to lack of KIR expression, almost all of the CD56^bright^ NK subset (>95%) expresses NKG2A/CD94 (**S4A Fig**). We also noted that among CD56^dim^ peripheral blood NK cells, 35% expressed NKG2A/CD94, 20% expressed KIR2DL1, 25% expressed KIR2DL2/3 and 17% expressed KIR3DL1 (**S4A Fig**). Based on the data presented in Figs 1 and 2 and differences in the expression of specific iNKRs on CD56^dim^ and CD56^bright^ NK cells, we posit that CD56^bright^ NK cells have a greater capacity to degranulate against HIV-infected cells than CD56^dim^ NK cells. We found that a higher proportion of CD56^bright^ NK cells than CD56^dim^ NK cells degranulated, when exposed to autologous HIV-infected T-cells (**Fig 3A and 3B**). This difference was not limited to the clone of HIV-1 used to infect the target cells as we found similar levels of degranulation from CD56^bright^ NK cells exposed to T-cells infected with three different strains of HIV-1 (**S4B Fig**).

In most individuals, about 10% of peripheral blood NK cells are CD56^bright^, whereas the vast majority of NK cells (≥90%) are CD56^dim^. We therefore tested, on a per cell basis, how well CD56^bright^ NK cells degranulated in response to HIV-infected T-cells relative to CD56^dim^ NK cells. For this study, we utilized primary NK cells sorted based on differential expression of CD56 (**S4C Fig**). When purified CD56^bright^ NK cells were co-cultured with HIV-infected T-cells, we noted at least a 2-fold increase in their capacity to degranulate as compared to purified CD56^dim^ NK cells exposed to the same target cells (**Fig 3C**).

Previous investigations claim that CD56^dim^ NK cells have a greater cytotoxic potential as compared to CD56^bright^ NK cells [35,36], and that CD56^bright^ NK cells have lower levels of perforin and granzymes to lyse target cells, as compared with CD56^dim^ NK cells [37]. In our studies, all CD56^bright^ NK cells expressed perforin, albeit at 2-fold lower levels compared with CD56^dim^ NK cells (**S4D Fig**). Moreover, we found lower levels of granzyme-A and -B, but higher average levels of granulysin and granzyme-K in the CD56^bright^ subset (**S4E and S4F Fig**). IL-2 treatment of CD56^bright^ NK cells appear to enhance the overall expression of perforin and granzyme B but did little to enhance Granzyme K or granulysin expression (**S4G Fig**). Therefore, CD56^bright^ NK cells indeed possess the appropriate cytolytic granule components to effectively mediate cytotoxicity.

Based on these findings, we tested the degree to which CD56^bright^ NK cells lysed HIV-infected T-cells as compared to CD56^dim^ NK cells. On a per cell basis, purified CD56^bright^ NK cells had at least a 2-fold greater capacity to lyse HIV-infected cells than CD56^dim^ NK cells (**Fig 3D**). In conclusion, CD56^bright^ NK cells are able to kill HIV-infected cells to a greater degree, on a per cell basis, than CD56^dim^ NK cells.

### In response to HIV-infected T-cells, NKG2A/CD94^+^ KIR2DL^-^ CD56^dim^ NK cells degranulate comparably to the CD56^bright^ subset

**Fig 4:**
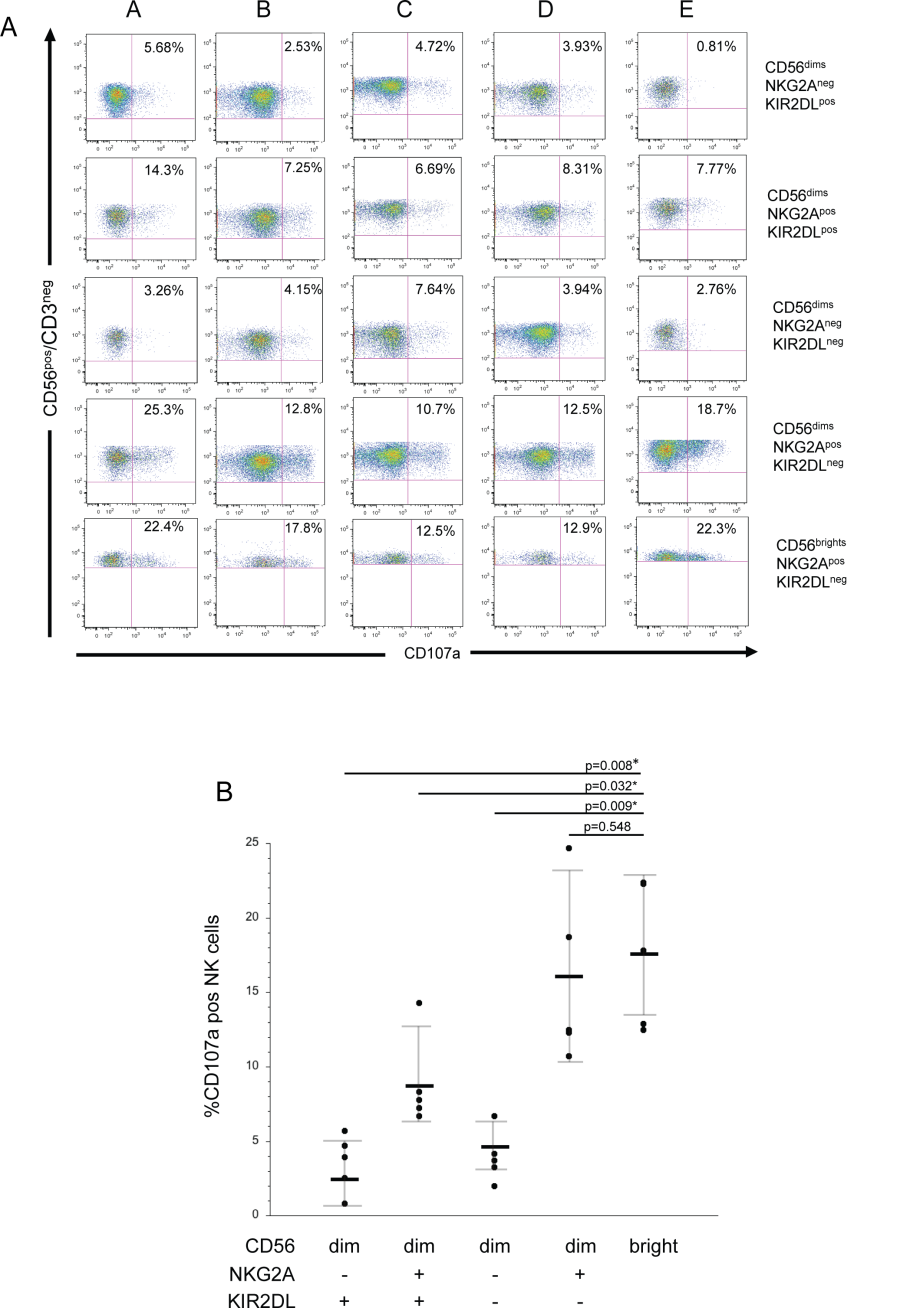
CD56^dim^ NK cells expressing NKG2A/CD94 but lacking KIR2DLs degranulate similarly to CD56^bright^ NK cells in response to autologous HIV-infected T-cells. (A) Degranulation of the CD56^dim^ NK cell subset from five different donors based on their expression of NKG2A/CD94 and/or KIR2DL in response to HIV-infected T-cells (five subjects A-E). The response of CD56^bright^ NK cells to the same target cells is also given. (B) Summary of the percent degranulation by CD56^bright^ NK cells and the various CD56^dim^ NK cell subpopulations in response to autologous HIV-infected T-cells from five different donors. Bars represent the mean of the CD107a responses of each NK cell subpopulation of the different donors. Gray error bars represent the standard deviation of the mean Statistical significance (p≤0.05) of the differences in responses was determined using the Wilcoxon-ranked sum test.

Since the vast majority of CD56^bright^ NK cells express NKG2A/CD94 and lack KIR2DLs (**S4A Fig**), the CD56^bright^ NK cells are less likely to be negatively affected by HLA-C on the HIV-infected cell surface (**Fig 2**). Moreover, NKG2A/CD94 expression confers a greater capacity to degranulate against HIV-infected cells since HLA-E does not appear to effectively engage the iNKR (**Fig 1**). Although we did not observe an increased frequency of NKG2D on CD56^bright^ NK cells compared to CD56^dim^ NK cells, we did note an increased presence of the receptor on CD56^bright^ NK cells relative to CD56^dim^ NK cells (**S4H Fig**). Thus, we tested whether CD56^dim^ NK cells expressing NKG2A/CD94 and lacking KIR2DLs have a similar capacity to degranulate as CD56^bright^ NK cells. We gated the CD56^dim^ NK cells into four possible combinations: (i) NKG2A/CD94^-^ KIR2DL^+^, (ii) NKG2A/CD94^+^ KIR2DL^+^, (iii) NKG2A/CD94^-^ KIR2DL^-^ and (iv) NKG2A/CD94^+^ KIR2DL^-^ (**Fig 4A)**. Only the CD56^dim^ NKG2A/CD94^+^ KIR2DL^-^ NK cell subpopulation showed a similar ability to the CD56^bright^ NK cell subpopulation to degranulate in response to HIV-infected cells (**Fig 4B)**. Taken together, our findings indicate that cytotoxic responses to HIV-infected T-cells are highest among NK cells lacking KIR2DLs and expressing NKG2A/CD94 compared to all other NK subpopulations.

## Discussion

Our main findings are that the NK cell subset most likely to respond to HIV-infected T-cells expresses NKG2A/CD94 and that HLA-E on the infected cell does not effectively engage with this iNKR. This conclusion is supported by several lines of evidence from the current study: 1) More than twice as many NKG2A/CD94^+^ NK cells released the contents of their lytic granules when exposed to HIV-infected cells compared with NK cells lacking this iNKR; 2) using an anti-iNKR Ab to block NKG2A/CD94 from interacting with HLA-E had only minimal effects on the ability of the responding NKG2A/CD94^+^ NK cells to degranulate in response to HIV-infected cells. In contrast, the same anti-NKG2A blocking antibody increased degranulation of NKG2A/CD94^+^ NK cells in response to a B-cell line expressing HLA-E. NKG2A/CD94^+^ and not NKG2A/CD94^-^ memory NK cells are cytolytic to target cells expressing Gag-specific peptides [38]. Our findings are also aligned with studies by investigators indicating that primary NK cell population in SIV-infected primates that are cytolytic express NKG2A/CD94 [38,39]. One possible explanation for the greater ability of NKG2A/CD94^+^ NK cells to degranulate compared with NKG2A/CD94^-^ NK cells may lie in differences in activation receptors expressed on the two subsets. One of the critical NK cell activation receptors involved in triggering NK cell lysis of HIV-infected cells is NKG2D [40]. We did not find any difference in the frequency of NKG2D^+^ NK cells possessing or lacking NKG2A/CD94.

We also found that NK cells expressing NKG2C/CD94, an HLA-E-specific activating receptor, are not responsible for enhanced NK cell responsiveness toward HIV-infected cells. The outcome of our study could be due to the inability of the NKG2C/CD94^+^ NK cells to recognize HLA-E on HIV-infected T-cells or to a greater restriction of NKG2C/CD94^+^ NK lytic activity through inhibition by HLA-C. In support of the latter possibility, we found a higher proportion of NKG2C/CD94^+^ NK cells expressing HLA-C-specific iNKRs (*i*.*e*., KIR2DLs).

A major question raised by our study is: Why are NKG2A/CD94^+^ NK cells likely to lyse HIV-infected cells compared to NK cells lacking this iNKR, despite the presence of HLA-E on infected cells? One possibility is that HIV induces the release of heat-shock proteins (HSP) from the mitochondria [41], which have been shown to interact with HLA-E on target cells but not engage NKG2A/CD94 on NK cells [13]. However HIV induces the release of HSP in HIV-infected cells through the actions of Vpr and not Gag [41]. Based on our data, we posited that a conserved peptide AISPRTLNA (AA9) derived from the HIV-1 Gag (*i*.*e*. capsid protein) is presented by HLA-E on HIV-infected cells [17], making it unrecognizable by NKG2A/CD94. We are keenly aware that our findings are not consistent with the current paradigm, which holds that a monomorphic leader peptide nonamer derived from HLA-A, -B, -C or -G is normally present within the binding groove of HLA-E [42,43]. However, peptides other than the leader peptides of MHC-I can be presented by HLA-E [11,12]. We showed in the present study that a highly conserved HIV capsid peptide, AA9, presented by HLA-E, is unable to interact with NKG2A/CD94 because it possesses an asparagine in position 8 rather than a large hydrophobic residue, as found in peptides capable of interacting with CD94 specifically [7]. When the asparagine residue was replaced with a phenylalanine within the HIV capsid peptide, NK cells expressing NKG2A/CD94 lost their capacity to degranulate when exposed to autologous HIV-infected T-cells. We further showed in this study that AA9 and N-extended precursors are produced in the cytosol of activated CD4 T cells, in support of their possible presentation by HIV-infected cells.

While the direct identification of AA9 from HIV-infected cells remains challenging, the amount of AA9 and N-extended peptides produced in the cytosol compared to other known HIV epitopes in this fragment would be sufficient for presentation and detection by immune cells [30,31]. Moreover, we showed that HLA-E tetramers possessing AA9 bind to NK cells 3-fold less effectively than HLA-E tetramer loaded with the nonamer HIV capsid peptide with a phenylalanine in position 8. Moreover, blocking CD94 prevented HLA-E tetramer with AISPRTLFA from binding CD56^bright^ NK cells. Taken together, these findings led us to hypothesize that this non-MHC-I leader peptide derived from HIV-1 is efficiently presented by HLA-E on infected cells and blocks the tolerizing interaction with NKG2A/CD94. While alternative explanations for these data are indeed possible, if our hypothesis is correct, it would suggest that HIV, quite surprisingly, has not evolved to overcome this mechanism that potentiates NK cell attack of infected cells. Instead, this capsid peptide sequence is highly conserved in HIV-1, since it may be critical for viral replication fitness [32]. In fact, we found the same effect using three isolates of HIV-1 and multiple human donors. Despite the inherent advantage of NKG2A/CD94 subset of NK cells in responding to HIV-1 infection, we speculate that HIV may have evolved other means to ultimately avoid NK cell destruction [*e*.*g*., by down-modulation of activation ligands [44]].

Unlike the failure of HLA-E to inhibit NK cell cytolytic responses to HIV-1-infected T-cells, the negative impact of HLA-C on NK cytotoxic responses to autologous HIV-infected cells is consistent with predictions from previous studies that showed that the selective retention of HLA-C on HIV-infected T-cells prevents NK cell responses [3,45]. In support of this conclusion, we found that treatment of NK cells with blocking antibodies to the HLA-C-specific iNKRs (KIR2DL) enhanced NK cell degranulation toward HIV-infected T-cells. Moreover, NK cells possessing KIR2DL receptors in the context of their cognate ligands are at least two-fold less likely to degranulate compared to NK cells lacking KIR2DL receptors. Despite the capacity of HLA-C to inhibit NK cell cytolytic responses, there are still significant numbers of NK cells lacking expression of KIR2DLs.

In the current study, we also show that CD56^bright^ NK cells, on a per cell basis, are better at lysing HIV-infected cells than CD56^dim^ cells. These observations are not consistent with the widely held belief that CD56^bright^ NK cells have minimal cytotoxic capability relative to CD56^dim^ NK cells [35,36,37]. CD56^bright^ NK cells are considered immature relative to CD56^dim^ NK cells since the CD56^bright^ cells contain longer telomeres and express c-Kit [46], markers that are widely associated with immature cells. We demonstrate here that CD56^bright^ NK cells contain considerable amounts of perforin, granzymes, and granulysin. We also found that IL-2 treatment enhanced the cytotoxic response of CD56^bright^ NK cells. Although IL-2 enhances CD56 expression on NK cells [47], in our studies, we treated the NK cells with IL-2 after sorting. Yet the CD56^bright^ NK cells still responded better to HIV-infected cells then CD56^dim^ NK cells, due to their high incidence of NKG2A/CD94 and lack of KIR2DL expression. The better response of CD56^bright^ NK cells is likely potentiated by the increased sensitivity of these cells to IL-2, since they selectively express the high affinity IL-2 receptor [48]. Moreover, the levels of perforin and granzyme-B in the CD56^bright^ NK cells relative to CD56^dim^ NK cells are elevated in IL-2 treated NK cells while, granulysin and of granzymes-A, and -K, are similar within NK cells treated with IL-2.

The observation that, overall, CD56^bright^ NK cells are better at lysing HIV-infected cells is consistent with their predominant expression of NKG2A/CD94 and relative lack of KIR expression. In contrast, CD56^dim^ NK cells are less likely to express NKG2A/CD94, and express higher frequencies of KIR2DL receptors, which are negatively affected by HLA-C on the HIV-infected cell surface. This view is supported by our findings that CD56^dim^ NK cells are just as capable of degranulating in response to HIV-infected T-cells as CD56^bright^ NK cells if the CD56^dim^ NK cells express NKG2A/CD94 and lack KIR2DLs. Although, it would be desirable to isolate the various CD56^dim^ subsets based on iNKR expression to compare their capacity to kill HIV-infected cells in a cytolytic assay (*i*.*e*., ^51^Cr release assay), most KIR antibodies are not specific and can engage both inhibitory and activating KIR, and their binding to these receptors could alter NK cell biology by directly transducing activating or inhibitory signals, or through blocking of HLA ligand recognition.

Within HIV-infected individuals, the frequency of NK cells expressing KIR2DL receptors and NKG2A/CD94 is normal when the subject is aviremic [4,16]. However, as an infected person develops viremia, the percentage of NK cells expressing KIR2DL increases while the frequency of NK cells expressing NKG2A/CD94 declines [4,16]. Based on the findings in the present study, the KIR2DL^+^ NK cells in viremic subjects would likely be minimally responsive to HIV-infected T-cells. The shift in iNKR repertoire as viremia develops may be due, in part, to death of the HIV-responsive NKG2A/CD94^+^ NK cells that are highly cytolytic toward HIV-infected T-cells, resulting in their decline as they become overwhelmed by persistent responses to increasing viral infection. In contrast, HIV-infected cells retain HLA-C, resulting in an increased percentage of KIR2DL^+^ NK cells that are tolerized by HLA-C, which translates to a diminished capacity to degranulate when exposed to infected cells.

Even though the viral load is brought to undetectable levels in infected individuals soon after initiation of combined antiretroviral therapy, the recovery of NKG2A/CD94^+^ NK cells is relatively slow; taking up to two years to reach levels observed in healthy HIV-uninfected people [16]. Moreover, the incidence of NK cells expressing NKG2C/CD94 is relatively high within HIV-infected viremic subjects, most likely the result of human cytomegalovirus infection [49]. Unexpectedly, however, we found here that NKG2C^+^ NK cells do not have a greater capacity to degranulate in response to HIV-infected cells, further supporting the possibility that these NK cells are expanding in response to HCMV. Interestingly, viremic individuals exhibit roughly a 2-fold increase in the percentage of NK cells that express KIR3DL1, as compared to aviremic subjects. Based on our studies and those of others [20], this would be expected to result in a population of NK cells that controls HIV effectively. However, as mentioned above, the percentage of NK cells expressing KIR2DL receptors is >2-fold higher in viremic subjects compared to aviremic individuals. Thus, the KIR3DL1^+^ NK cells are also more likely to express KIR2DLs and, as such, be less responsive to HIV-infected T-cells that retain HLA-C.

Taken together, our findings provide valuable new insights regarding the capacities of distinct NK cell subsets to respond to HIV-infected T cells. Further studies, including clinical ones, are clearly needed to confirm our findings and to determine the shifting ability of iNKRs to regulate the capacity of NK cells to control HIV replication in the context of an ongoing infection in patients. Understanding the mechanism leading to a loss of NKG2A/CD94 expressing NK cells and an increase in KIR-expressing NK cells in the context of HIV infection is needed, especially because these changes appear not to be caused by HIV [16]. Recently, it has been proposed to use NK cells to attack HIV-1 latently infected cells in the context of latency reversing agents [50]. However, even after antiretroviral therapy, only minimal changes occur in the distribution of iNKR on NK cells [16], indicating their ability to control HIV in patients treated with latency reversing drugs may be limited. Our results provide a mechanistic basis to guide these future clinical studies and suggest that providing patients with ex vivo-expanded populations of NKG2A/CD94^+^ KIR2DL^-^ NK cells may be uniquely beneficial.

## Materials and Methods

### Ethical statement

All primary cells (i.e., NK cells and CD4^+^ T-cells) used in this study were isolated from peripheral blood of healthy HIV-1 uninfected donors and two HIV-infected donors after informed written consent was acquired in accordance with the Declaration of Helsinki. The Institutional Review Board at Rush University Medical Center under protocol 11010703-IRB01 (Chicago, IL, USA) approved the study protocol.

PBMC used at Mass. General Hospital for peptide degradation assays were isolated from buffy coats collected from anonymous blood donors as approved by the Partners Human Research Committee under protocol 2005P001218 (Boston, MA USA). CD4 T cells were isolated and stimulated as described below.

### Primary cells and cell lines

NK cells and CD4^+^ T-cells were isolated from the peripheral blood in separate blood draws and stimulated *in vitro* as described by Davis et al [51]. All uninfected donors were genotyped for their HLA alleles by the HLA Laboratory at Rush University Medical Center.

The P815 mouse lymphoblast-like mastocytoma cell-line (ATCC) was maintained in DMEM medium supplemented with 10% heat inactivated (56°C, 30 min) fetal bovine serum (FBS) and penicillin/streptomycin (Mediatech). K562 erythromyleoblastoid leukemia cell-line (ATCC) was maintained in RPMI-1640 medium (Mediatech) supplemented with 10% heat inactivated (56°C, 30 min) FBS and penicillin/streptomycin (RPMI complete medium). The Raji Burkitt’s lymphoma cell line (ATCC), 721.221 MHC-I-deficient EBV-transformed B cell line, and 721.221-Cw3, which was transfected with HLA-Cw3 cDNA, were maintained in RPMI-complete medium supplemented with 50 mM HEPES and 50 μM β-mercaptoethanol.

### HIV-infection of primary CD4^+^ T-cells

Freshly isolated primary CD4^+^ T-cells were activated using anti-CD3/anti-CD28 mAb coupled to magnetic beads (Miltenyi Biotech) or PHA (3 μg/mL) for 72 h prior to infection with an HIV-1 strain in which the HIV-1 envelope is deleted. These envelope-defective viruses were VSV-G pseudotyped. In our study, we also infected CD4^+^ T-cells with a molecular HIV clone (HIV-1_NL4/3_), primary isolates (HIV-1 _SHM-1_), and lab-adapted strains (HIV-1_SF162_).

The N153F mutation in Gag was created using the Lightning Site-Directed Mutagenesis Kit (Agilent Tech) according to the manufacturer’s protocol. The primers used were:

5’- GGTACATCAGGCCATATCACCTAGAACTTTATTTGCATGGGTAAAAGTA-3’ and 5’-TAC TTTTAC CCATGCAAATAAAGTTCTAGGTGATATGGCCTGAT-GTACC-3’. Because the mutation will not lead to viable capsid proteins in the virus, we provided a capsid for the virus particle in *trans* using the ΔR8.2 packaging vector as previously described [52].

Infection was performed by spin-inoculation with a TCID_50_ = 1 as described by O’Doherty et al [53]. Following infection, cells were cultured in RPMI complete medium with 200 U/mL recombinant IL-2 (AIDS Research and Reference Reagent Program, Division of AIDS, NIAID, NIH, deposited by Dr. Maurice Gately, Hoffmann-La Roche Inc, Nutley, NJ).

### Flow cytometry reagents and antibodies

Before staining surface markers, all samples were incubated with LIVE/DEAD Fixable Dead Cell Stain Kit (Invitrogen) per the manufacturer’s instructions. Fluorophore-conjugated antibodies were: CD3-PacificBlue (clone: UCHT1), CD14-PacificBlue (clone: M5E2), CD20-PacificBlue (clone: 2H7), CD56-AF700-APC (clone: HCD56), CD94-FITC (clone: DX22), CD158e1-FITC (clone: DX9), HLA-E-PE (clone: 3D12), CD107a-FITC, -PE Cy7 or -APC (clone: H4A3) (Biolegend), CD158a-PerCP Cy5.5 (clone: HP-MA4) (eBioscience), CD159a-PE (clone: Z199), CD158e1/e2-PE (clone: Z27.3.7) (Beckman Coulter), CD158b-APC (clone: DX27) (Miltenyi Biotech), Recombinant Human KIR2DL2/CD158b1 Human-IgG1-Fc Chimera (R&D systems), APC F(ab')_2_ Fragment Goat anti-Human IgG (Jackson Immuno), Biotinylated anti-HLA-Bw4, Biotinylated anti-HLA-Bw6 (One Lambda), Streptavadin-PE (Biolegend), anti-NKG2C-AF488 (clone: 134591) (R&D Systems), and Mouse IgG1. Before intracellular staining, all samples were permeabilized using Cytoperm/Cytofix (BD) per the manufacturer’s instructions. Antibodies for intracellular staining were: anti-perforin-FITC (clone: δG9) (BD), Granzyme A-FITC (clone: CB9), Granzyme B-FITC (clone: DH2), Granulysin-PE (clone: GB11) (Biolegend) and Granzyme K-PE (clone: GHM6C3) (Santa Cruz). Samples were collected on FACS_LSRII_ (BD Biosciences) and analyzed using FlowJo software (TreeStar). FACS_LSRII_ was a generous gift from the James B. Pendelton Charitable Trust.

The PE-conjugated HLA-E 0101 tetramers containing 100 nM of peptide 14–22 of the HIV-1_NL4/3_ capsid protein, the same 14–22 peptide of the HIV-1_NL4/3_ capsid protein with an F in place of the N, and the leader peptide for HLA-G, were produced at the NIH Tetramer Core Facility (Atlanta, GA). All three peptides were generated at the Protein Research Lab at the University of Illinois at Chicago.

### CD107a degranulation assay and chromium release assays

One day prior to the degranulation and cytotoxic assays, fresh NK cells were isolated from peripheral blood mononuclear cells (PBMCs) of the same donor who provided the CD4^+^ T-cells used to generate HIV-infected target cells. Purified NK cells were cultured overnight prior to exposing them to target cells. In ^51^Cr release assays purified NK cells were also cultured overnight in medium containing 200U/ml of IL-2. The PBMCs for isolation of NK cells were obtained from a separate blood draw that was done 6–9 days prior in order to obtain CD4^+^ T-cells. This procedure was used because it takes 7–10 days to stimulate and infect CD4^+^ T-cells with HIV-1. After isolation or sorting, NK cells were cultured overnight in either plain medium or in medium containing 200 U/ml IL-2. The resulting purified and cultured NK cells were placed in RPMI-1640 and 10% FBS and exposed to target cells.

HIV-infected cells were isolated from uninfected cells in bulk culture prior to addition to NK cells as described [51]. Briefly, HIV-infected were treated with anti-CD4 Ab coupled to magnetic beads at a ratio of ten beads per cell. The cells were incubated at 4°C for 1 hour. The cells bound to the beads were removed with a magnet and the remaining cells suspension treated with another round of anti-CD4 Ab coupled to magnetic beads for 1 hour at 4°C at a bead to cell ratio of 10:1. The resulting cells, after immunomagnetic bead separations of CD4+ T-cells, were shown to be infected by HIV as demonstrated by presence of HIV-1 p24 antigen within the unbound cells which was determined as described [51]. An example of HIV-infected remaining cells following 2 rounds of immunomagnetic removal of CD4^+^ T-cells is shown in **S1B Fig**. At the end of the remaining cell suspension was placed in medium added to the NK cells (at a ratio of 1 target cell to 2 NK cells) for the CD107a assay or labeled with ^51^Cr for 2 hours and added to the NK cell at an target to NK cell ratio of 1:1 or 1:3 for the ^51^Cr release assay. As control target cells, uninfected CD4+ T-cells (negative control) or K562 cells (positive control) were used. At the end of 4 hours of co-culture for the CD107a assay, NK cells were stained for CD107a as described in detail in [51]. In the case of ^51^Cr release assay culture fluid was collected and level of ^51^Cr in the fluid was determined as described in extensive detail in [51].

### Blocking interactions between NK cell receptors and their ligands

Prior to the addition of target cells, Fc receptors (CD16:FcγRIIIa) on NK cells were blocked at 4°C for 20 min with 15 μg/mL of the Fab fragment of anti-CD16 Ab (Ancell) in PBS. Cells were then washed 3 times in PBS to remove excess Fab fragment of the anti-CD16 Ab. Cells were blocked at 4°C for 20 min with 10 μg/mL of the following antibodies: anti-CD159a (clone:Z199) (Beckman Coulter), anti-CD158a (clone:143211), anti-CD158b (clone:180704) (R&D Systems), anti-CD158b (clone:DX27) (Biolegend), anti-CD158a (clone:HP-3E4) and anti-CD16 (clone:3G8) (BD). Rituxan (Genentech; 0.75 μg/mL) was used to trigger antibody-dependent cell-mediated cytotoxicity against Raji cells.

### Cytosolic degradation assays

Cytosolic extracts of CD3/28-activated CD4 T cells were prepared as described in [54]. 2 nmol of highly purified peptides were digested with 15μg of extracts at 37°C in degradation buffer [55]. The degradation was stopped with 2.5μL of 100% formic acid and peptide fragments were purified by 5% trichloroacetic acid precipitation. Degradation peptides were separated and identified by in-house LC-MS/MS using the conditions detailed in [31].

### Statistical analyses

Power analysis was used to determine the minimum number of individuals needed to provide 80% power to identify a statistically significant difference between two groups if there is one. The data in the study were analyzed non-parametrically using the Wilcoxon ranked-sum test or the Mann-Whitney U-test. All groups had an n ≥ 3. *p*-values ≤ 0.05 were considered statistically significant.

## Supporting Information

S1 Fig NKG2A/CD94^+^ NK cells degranulate in response to HIV-infected cells expressing HLA-E but not to a B-cell line expressing HLA-E. (A) The expression of HLA-E on HIV-infected primary T-cells [HIV-1 p24 antigen positive (blue line)], uninfected primary CD4^+^ T-cells (red line) and staining control (green line). This is an example of three separate experiments. (B) Example of purified HIV-infected cells (HIV-1 p24 antigen positive) following removal of CD4+ T-cells from HIV-infected bulk cultures. Results for the uninfected cells are also provided. Gates were set based on isotype staining controls for uninfected and infected cells. (C) NK cells stained for NKG2A or CD94 were evaluated for their ability to degranulate when exposed to HIV-infected T-cells. Bold numbers are percent of NKG2A^+^ or CD94^+^ (upper quadrants) and NKG2A^-^ or CD94^-^ (lower quadrants) NK cells that express CD107a on their surface after 4-hour exposure to HIV-infected T-cells. (D) Frequency of purified NK cells that are NKG2A/CD94 and/or NKG2C/CD94 positive. Value for NKG2A/CD94 and NKG2C/CD94 negative NK cells is given below the lower left quadrant. (E) Expression of KIR2DL-1 and/or -2/3 on NK cells expressing or lacking NKG2C. Bars represent mean frequency of NKG2C+ and NKG2C- NK cells expressing KIR2DLs of all subjects tested. (F) Ability of NK cell subsets expressing or lacking KIR3DL1 to degranulate in response to HIV-1 infected T-cells. NK cells and targets were derived from donors possessing at least one allele of MHC class I molecules with a HLA-Bw4 epitope (open circles) or two alleles of MHC class I molecules with a HLA-Bw6 (closed circles) epitope. Bars represents mean CD107a surface expression of NK cells following exposure to autologous HIV-infected cells for all donors in each group. Statistical significance (p≤0.05) of the differences was determined using the Wilcoxon-ranked sum test. (G) Ability of NK cells expressing or lacking KIR3DL1 that also lack KIR2DLs and NKG2A/CD94 to degranulate in response to HIV-1 infected T-cells. Statistical significance (p≤0.05) of the differences was determined using the Wilcoxon-ranked sum test. (H) Expression of HLA-E on 721.221 cells expressing HLA-Cw3 (blue line) and 721.221 cells (red line). Staining control (isotype control-green line) is also provided. (I) Percent CD107a expression by CD94 positive (black bars) or CD94 negative (white bars) NK cells lacking KIR2DL2 following exposure to 721.221 cells expressing HLA-Cw3 (KIR2DL2 ligand). Prior to adding the target cells the NK cells were blocked with either anti-CD16 Fab’ fragment alone or anti-NKG2A Ab and anti-CD16 Fab’ fragment. (J) Ability of anti-CD16 Fab’ fragment to inhibit antibody-dependent cell-mediated cytotoxicity of Rituximab-labeled Raji cell line by NK cells. Numbers in lower right quadrants represent the percent of CD56^dim^ NK cells that degranulated in response to antibody-labeled target cells. (K) Correlation of concentration of anti-CD16 Fab’ fragment and percent CD107a^+^ CD56^dim^ NK cells in response to Rituximab-labeled Raji cell line. (L) Ability of NK cells to degranulate after exposure to HIV-infected T-cells in the presence of blocking antibodies to NKG2A and anti-CD16 Fab’ fragment or anti-CD16 Fab’ fragment alone. (M) Ability of NK cells expressing and lacking NKG2A/CD94 from seven different donors to degranulate following exposure to K562 cells.
(PDF)

S2 Fig AISPRTLNA (AA9) and N-extended precursors can be produced during peptide degradation in activated CD4 T cells. (A) Presence of AISPRTLNA peptide sequence (highlighted in yellow) within the proteome of various HIV-1 strains. (B) Experimental design of the degradation of long peptides in cytosolic extracts from activated CD4 T cells. (C) Peptides generated during the degradation of 2-AA9-1 include remaining substrate 2-AA9-1 (grey), the epitope AA9 (blue), N-extended precursors (green), antitopes (orange). (D) Relative quantity of AA9 (blue) and N-extended AA9 produced during a 2-hour degradation of 2-AA9-1 (left) or of p24-10-35m (right) in cytosolic extracts of activated CD4 T cells from four healthy donors. N-extended AA9 correspond to 1- and 2-aa extended for 2-AA9-1 and up 3-AA9 for the 35-mer.
(PDF)

S3 Fig Impact of KIR2DL expression on NK cell responses to HIV-infected T-cells. (A) Percent of purified NK cells that are KIR2DL1 and/or KIR2DL2/3 positive. Value for KIR2DL1 and KIR2DL2/3 negative NK cells is given below the lower left quadrant. (B) KIR2DL (KIR2DL1 and KIR2DL2) positive and/or negative NK cells from a subject possessing HLA-C molecules with a lysine (C2) or asparagine (C1) in the 80^th^ position of the heavy chain. KIR2DL (KIR2DL1 and KIR2DL2) positive and/or negative NK cells expressing or lacking NKG2A/CD94 were evaluated for their ability to degranulate in response to CD4+ T-cells infected with HIV-1_NL4/3_, HIV-1_SF162_ or HIV-1_SMH1._ Percent of CD107a positive NK cells regardless of inhibitory receptor expression is also provided (unfractionated). Numbers in upper right quadrant are the percent CD107a positive NK cells following four-hour co-culture with HIV-infected T-cells. (C) Percent of CD107a positive NK cells expressing or lacking KIR3DL1, KIR2DLs or NKG2A/CD94 after a 4-hour exposure to HIV-infected T-cells. NK cells and CD4^+^ T-cells were acquired after informed consent from aviremic HIV-infected patients who have CD4 counts of 600/μl (patient 1) and 1000/μl of blood (patient 2) who have been on combined anti-retroviral therapy for >2 years. (D) CD107a expression on NK cell subsets of an HLA-Cw*1203 (C1) and Cw*1502 (C2) donor expressing or lacking KIR2DL1 and/or KIR2DL2/3 in response to K562 cells, uninfected CD4^+^ T-cells or HIV-infected primary T-cells. Response in the absence of targets is also shown. (E) Percent of CD107a positive purified NK cells after 4h incubation with anti-KIR2DL1 or anti- KIR2DL2/3 blocking Abs in the presence of an anti-CD16 Fab fragment and P815 cells. Frequency of CD107a positive NK cells in the absence of antibodies and/or anti-CD16 Fab fragment and P815 cells are provided as negative controls. As a positive control NK cells were labeled with anti-CD16 Ab and exposed to P815 cells.
(PDF)

S4 Fig Characteristics of CD56^dim^ and CD56^bright^ natural killer cells. (A) Expression of NKG2A, KIR2DL1, KIR3DL1 or KIR2DL2/3 on CD56^bright^ and CD56^dim^ NK cells. Data from nine different subjects are shown. Statistical significance (p≤0.05) of the differences in percent of CD56^brights^ and CD56^dims^ to express the various iNKRs was determined using the Wilcoxon-ranked sum test. Bars represent mean frequency of NK cells in each subset expressing the indicated inhibitory receptor of nine different subjects. (B) CD56^bright^ NK cells are able to degranulate to a greater extent compared to CD56^dim^ NK cells when exposed to T-cells infected with HIV-1_NL4/3_, HIV-1_SF162_ or HIV-1_SMH1._ CD107a expressed on NK cells following exposure to uninfected T-cells and K562 cells are also provided for control target cells. Numbers in upper right quadrant are the percent CD107a positive NK cells following four-hour co-culture with target cells. (C) Gating strategy for sorting CD56^dim^ and CD56^bright^ NK cells from two subjects. (D) Expression of intracellular perforin in purified NK cells. Perforin levels within B-cells are also provided. (E) Expression of intracellular granzyme A, B and K in purified NK cells. (F) Expression of intracellular granulysin in purified NK cells. (G) Mean ratio of intracellular perforin, granzymes (-A, -B & -K) and granulysin within untreated and IL-2-treated CD56^bright^ NK cells compared to untreated and IL-2-treated CD56^dim^ NK cells of five different subjects. Error bars represents standard deviation of the mean ratio. (H) Frequency and median fluorescent intensity of NKG2D expression on CD56^bright^ (dark bars) and CD56^dim^ (white bars) NK cells of three different donors.
(PDF)
