# Supplementary figures and images for: A Conserved HIV-1-Derived Peptide Presented by HLA-E Renders Infected T-cells Highly Susceptible to Attack by NKG2A/CD94-Bearing Natural Killer Cells
Source: PLoS Pathog. 2016 Feb 1;12(2):e1005421. doi: 10.1371/journal.ppat.1005421 (PMC4735451; doi:10.1371/journal.ppat.1005421)

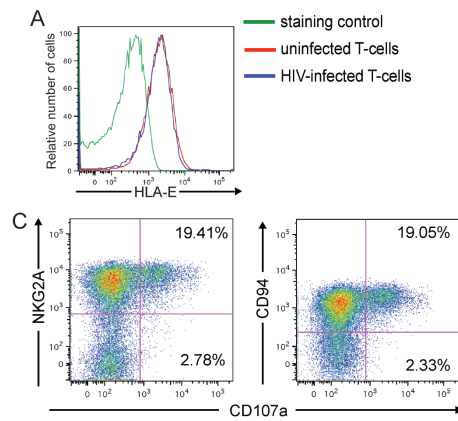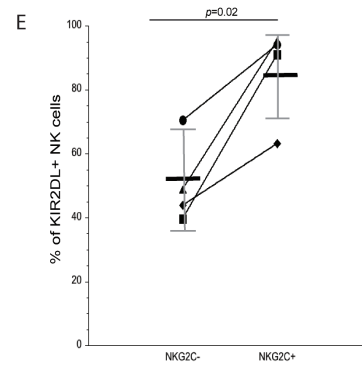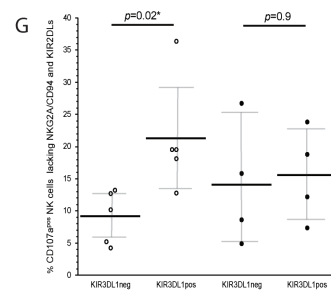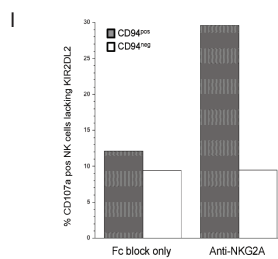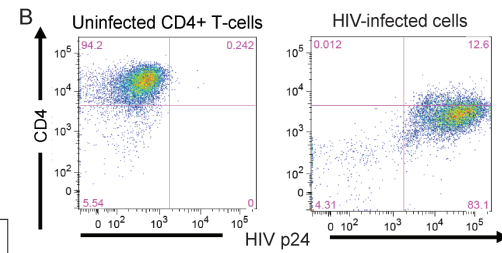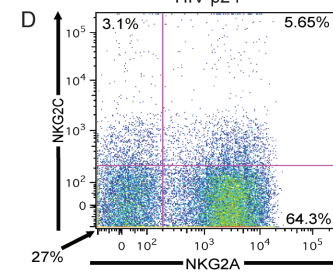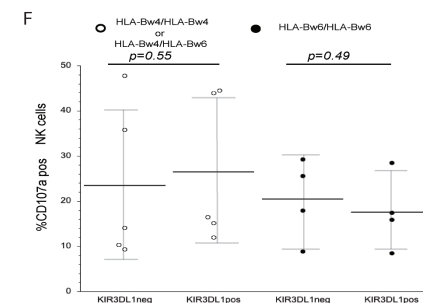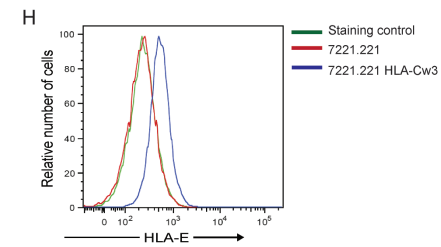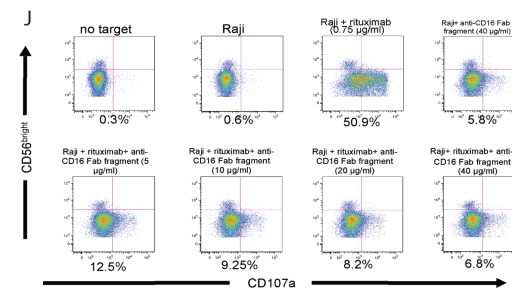

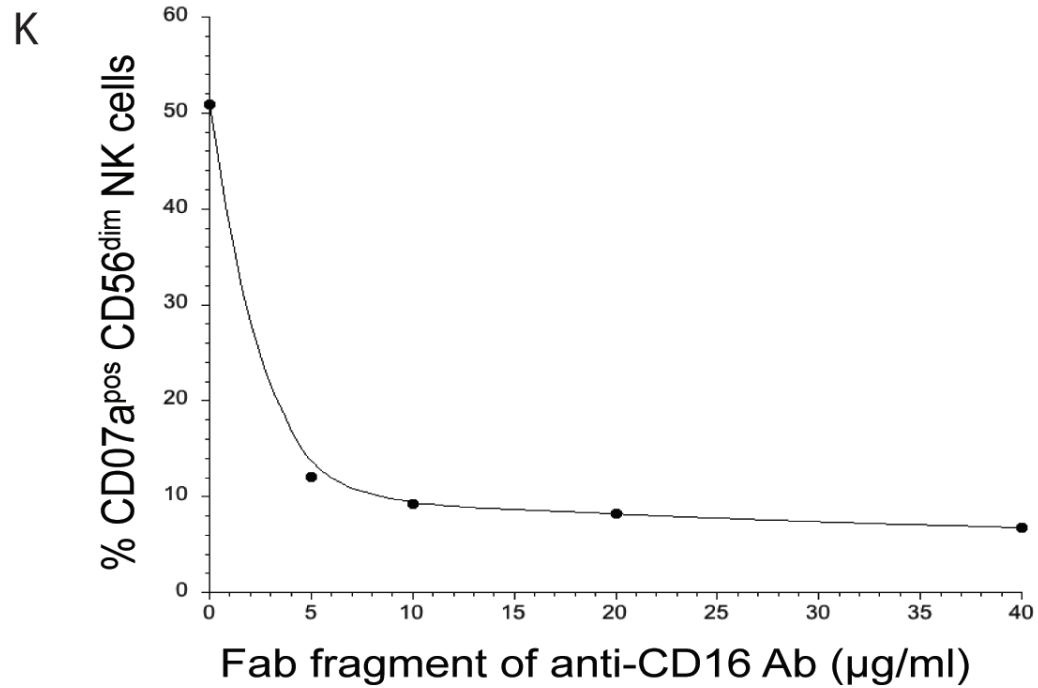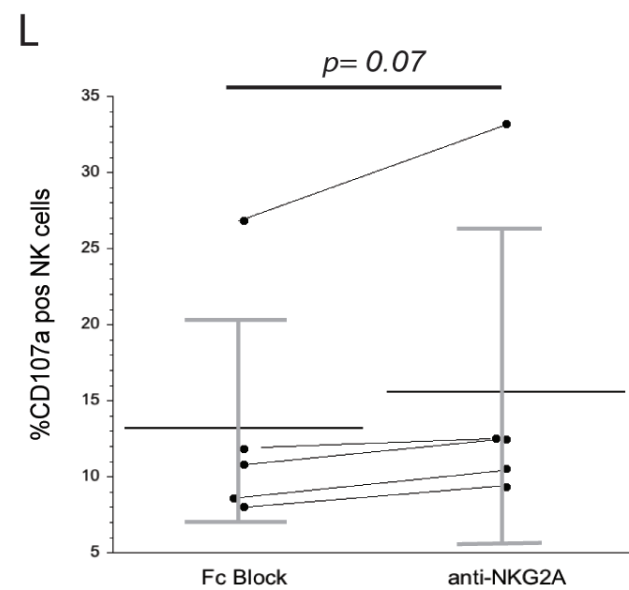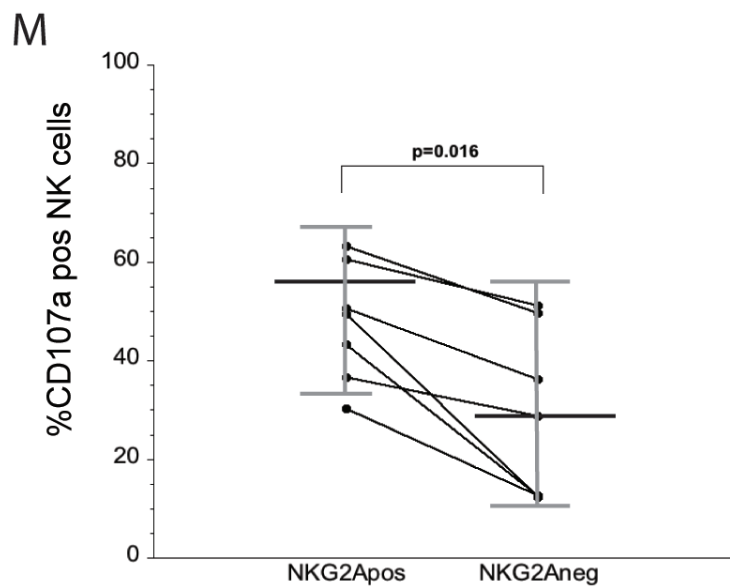

Supplement: S1 Fig — (A) The expression of HLA-E on HIV-infected primary T-cells [HIV-1 p24 antigen positive (blue line)], uninfected primary CD4+ T-cells (red line) and staining control (green line). This is an example of three separate experiments. (B) Example of purified HIV-infected cells (HIV-1 p24 antigen positive) following removal of CD4+ T-cells from HIV-infected bulk cultures. Results for the uninfected cells are also provided. Gates were set based on isotype staining controls for uninfected and infected cells. (C) NK cells stained for NKG2A or CD94 were evaluated for their ability to degranulate when exposed to HIV-infected T-cells. Bold numbers are percent of NKG2A+ or CD94+ (upper quadrants) and NKG2A- or CD94- (lower quadrants) NK cells that express CD107a on their surface after 4-hour exposure to HIV-infected T-cells. (D) Frequency of purified NK cells that are NKG2A/CD94 and/or NKG2C/CD94 positive. Value for NKG2A/CD94 and NKG2C/CD94 negative NK cells is given below the lower left quadrant. (E) Expression of KIR2DL-1 and/or -2/3 on NK cells expressing or lacking NKG2C. Bars represent mean frequency of NKG2C+ and NKG2C- NK cells expressing KIR2DLs of all subjects tested. (F) Ability of NK cell subsets expressing or lacking KIR3DL1 to degranulate in response to HIV-1 infected T-cells. NK cells and targets were derived from donors possessing at least one allele of MHC class I molecules with a HLA-Bw4 epitope (open circles) or two alleles of MHC class I molecules with a HLA-Bw6 (closed circles) epitope. Bars represents mean CD107a surface expression of NK cells following exposure to autologous HIV-infected cells for all donors in each group. Statistical significance (p≤0.05) of the differences was determined using the Wilcoxon-ranked sum test. (G) Ability of NK cells expressing or lacking KIR3DL1 that also lack KIR2DLs and NKG2A/CD94 to degranulate in response to HIV-1 infected T-cells. Statistical significance (p≤0.05) of the differences was determined using the Wilcoxon-ra [file ppat.1005421.s001.pdf]

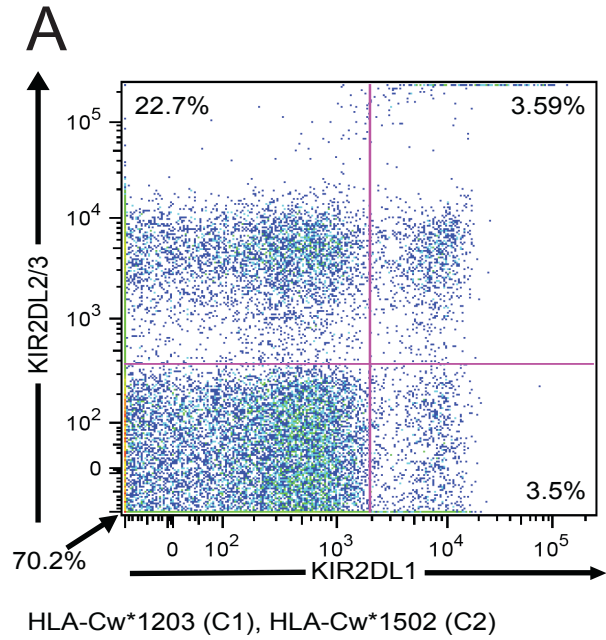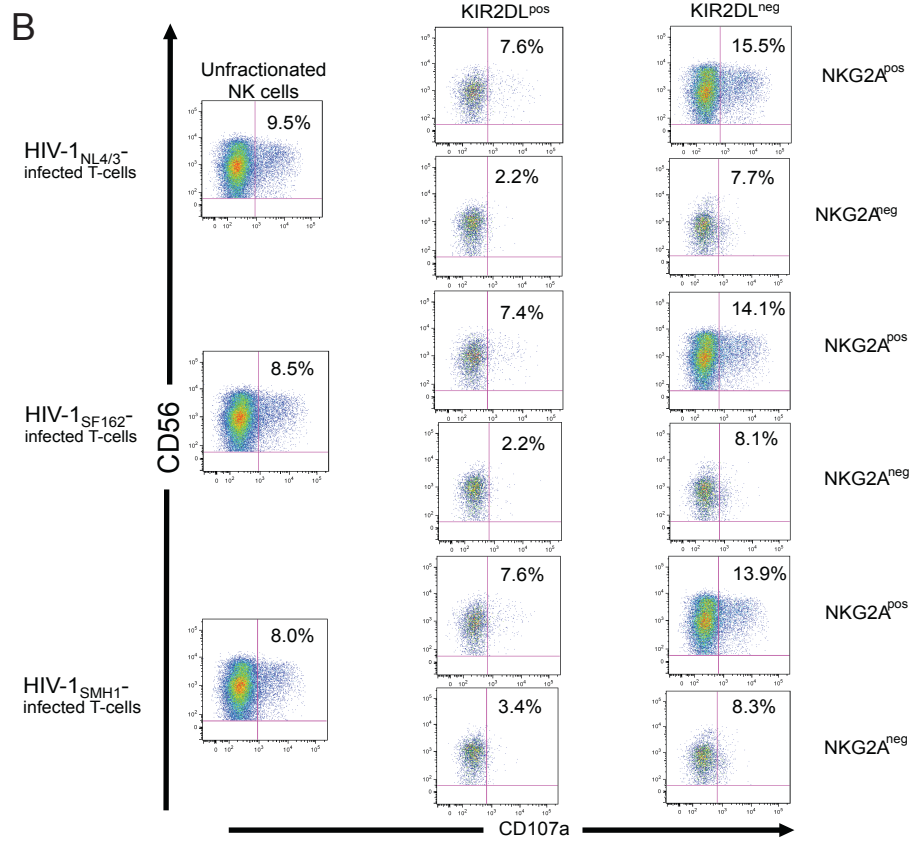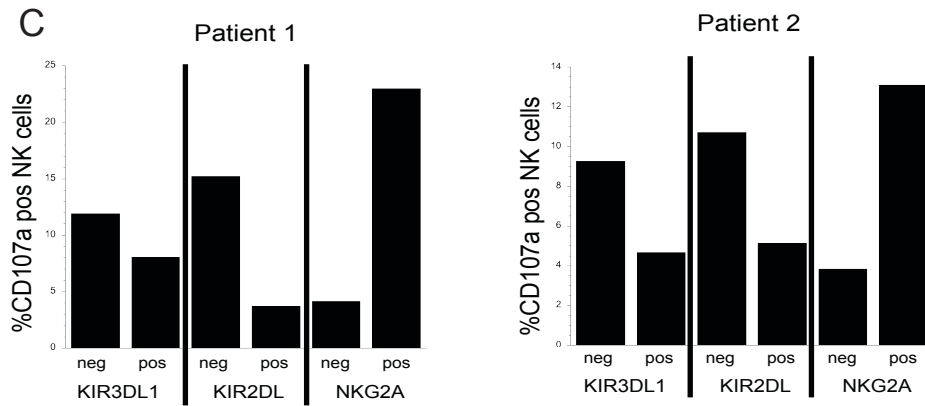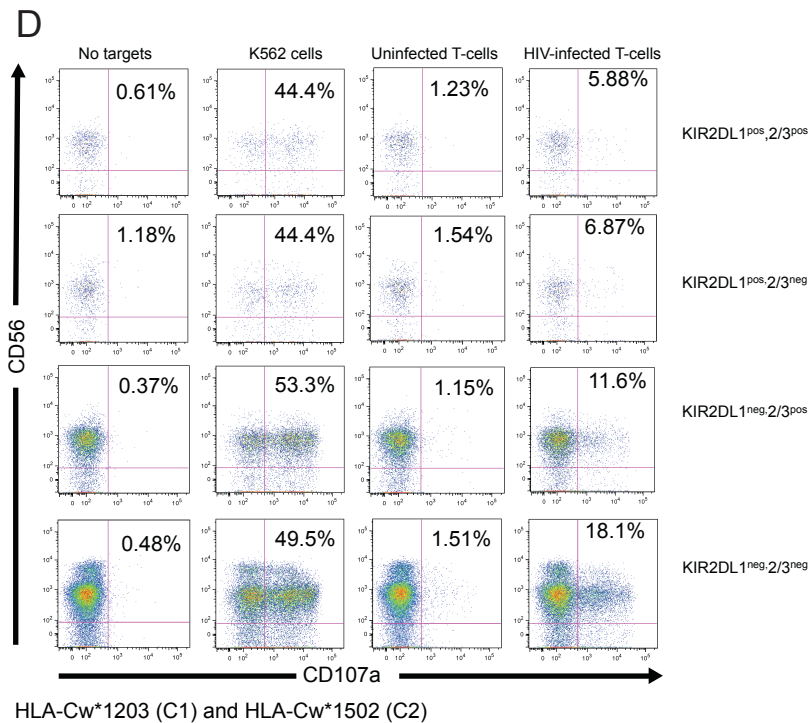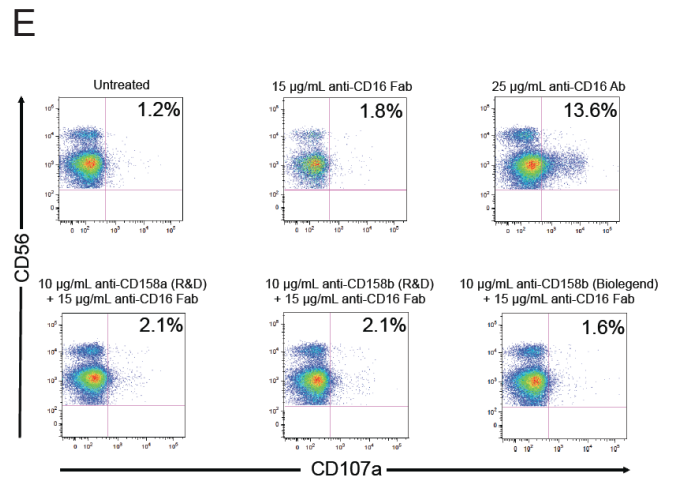

Supplement: S3 Fig — (A) Percent of purified NK cells that are KIR2DL1 and/or KIR2DL2/3 positive. Value for KIR2DL1 and KIR2DL2/3 negative NK cells is given below the lower left quadrant. (B) KIR2DL (KIR2DL1 and KIR2DL2) positive and/or negative NK cells from a subject possessing HLA-C molecules with a lysine (C2) or asparagine (C1) in the 80th position of the heavy chain. KIR2DL (KIR2DL1 and KIR2DL2) positive and/or negative NK cells expressing or lacking NKG2A/CD94 were evaluated for their ability to degranulate in response to CD4+ T-cells infected with HIV-1NL4/3, HIV-1SF162 or HIV-1SMH1. Percent of CD107a positive NK cells regardless of inhibitory receptor expression is also provided (unfractionated). Numbers in upper right quadrant are the percent CD107a positive NK cells following four-hour co-culture with HIV-infected T-cells. (C) Percent of CD107a positive NK cells expressing or lacking KIR3DL1, KIR2DLs or NKG2A/CD94 after a 4-hour exposure to HIV-infected T-cells. NK cells and CD4+ T-cells were acquired after informed consent from aviremic HIV-infected patients who have CD4 counts of 600/μl (patient 1) and 1000/μl of blood (patient 2) who have been on combined anti-retroviral therapy for >2 years. (D) CD107a expression on NK cell subsets of an HLA-Cw*1203 (C1) and Cw*1502 (C2) donor expressing or lacking KIR2DL1 and/or KIR2DL2/3 in response to K562 cells, uninfected CD4+ T-cells or HIV-infected primary T-cells. Response in the absence of targets is also shown. (E) Percent of CD107a positive purified NK cells after 4h incubation with anti-KIR2DL1 or anti- KIR2DL2/3 blocking Abs in the presence of an anti-CD16 Fab fragment and P815 cells. Frequency of CD107a positive NK cells in the absence of antibodies and/or anti-CD16 Fab fragment and P815 cells are provided as negative controls. As a positive control NK cells were labeled with anti-CD16 Ab and exposed to P815 cells. (PDF) [file ppat.1005421.s003.pdf]

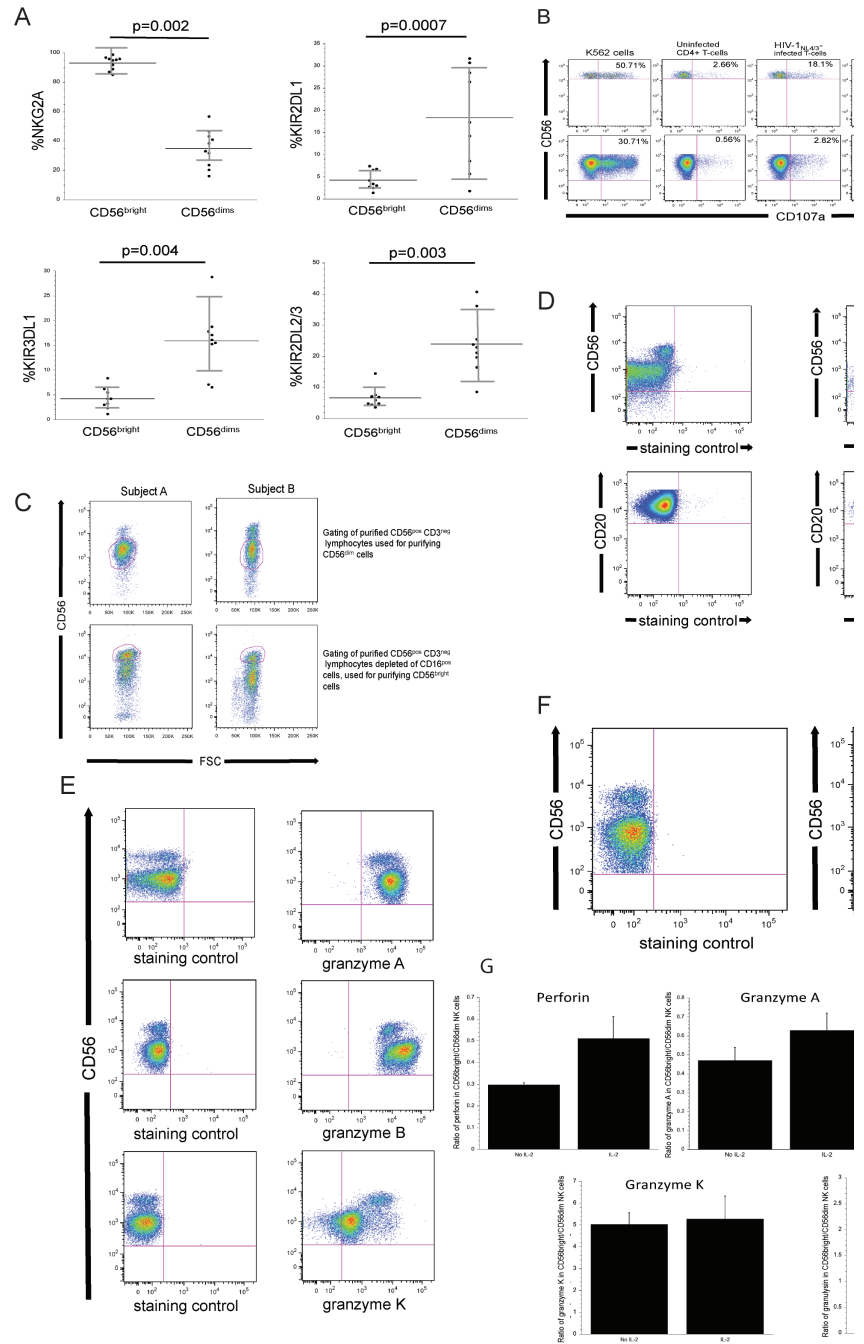

H

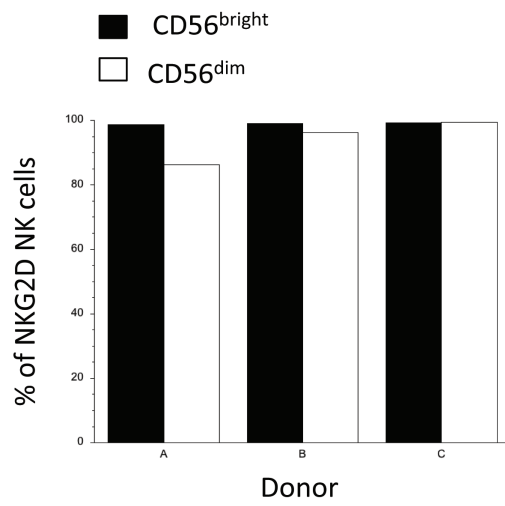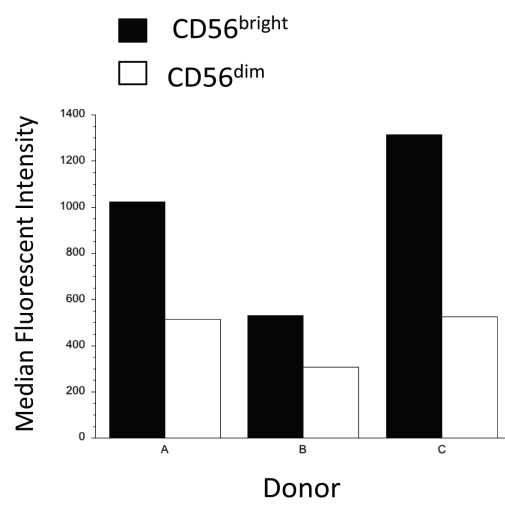

Supplement: S4 Fig — (A) Expression of NKG2A, KIR2DL1, KIR3DL1 or KIR2DL2/3 on CD56bright and CD56dim NK cells. Data from nine different subjects are shown. Statistical significance (p≤0.05) of the differences in percent of CD56brights and CD56dims to express the various iNKRs was determined using the Wilcoxon-ranked sum test. Bars represent mean frequency of NK cells in each subset expressing the indicated inhibitory receptor of nine different subjects. (B) CD56bright NK cells are able to degranulate to a greater extent compared to CD56dim NK cells when exposed to T-cells infected with HIV-1NL4/3, HIV-1SF162 or HIV-1SMH1. CD107a expressed on NK cells following exposure to uninfected T-cells and K562 cells are also provided for control target cells. Numbers in upper right quadrant are the percent CD107a positive NK cells following four-hour co-culture with target cells. (C) Gating strategy for sorting CD56dim and CD56bright NK cells from two subjects. (D) Expression of intracellular perforin in purified NK cells. Perforin levels within B-cells are also provided. (E) Expression of intracellular granzyme A, B and K in purified NK cells. (F) Expression of intracellular granulysin in purified NK cells. (G) Mean ratio of intracellular perforin, granzymes (-A, -B & -K) and granulysin within untreated and IL-2-treated CD56bright NK cells compared to untreated and IL-2-treated CD56dim NK cells of five different subjects. Error bars represents standard deviation of the mean ratio. (H) Frequency and median fluorescent intensity of NKG2D expression on CD56bright (dark bars) and CD56dim (white bars) NK cells of three different donors. (PDF) [file ppat.1005421.s004.pdf]
