# Supplementary material for: A Conserved HIV-1-Derived Peptide Presented by HLA-E Renders Infected T-cells Highly Susceptible to Attack by NKG2A/CD94-Bearing Natural Killer Cells
Source: PLoS Pathog. 2016 Feb 1;12(2):e1005421. doi: 10.1371/journal.ppat.1005421 (PMC4735451; doi:10.1371/journal.ppat.1005421)

A

```
Group M Virus (NL4.3)

MGARASVLSG GELDKWEKIR LRPGGKKQYK LKHIVWASRE LERFAVNPGL LETSEGCRQI
LGQLQPSLQT GSEELRSLYN TIAVLYCVHQ RIDVKDTKEA LDKIEEEQNK SKKKAQQAAA
DTGNNSQVSQ NYPIVQNLQG QMVHQAISPR TLNAWVKVVE EKAFSPEVIP MFSALSEGAT
PQDLNTMLNT VGGHQAAMQM LKETINEEAA EWDRLHPVHA GPIAPGQMRE PRGSDIAGTT
STLQEQIGWM THNPPIPVGE IYKRWIILGL NKIVRMYSPT SILDIRQGPK EPFRDYVDRF
YKTLRAEQAS QEVKNWMTET LLVQNANPDC KTILKALGPG ATLEEMMTAC QGVGGPGHKA
RVLAEAMSQV TNPATIMIQK GNFRNQRKTV KCFNCGKEGH IAKNCRAPRK KGCWKCGKEG
HQMKDCTERQ ANFLGKIWPS HKGRPGNFLQ SRPEPTAPPE ESFRFGEETT TPSQKQEPID
KELYPLASLR SLFGSDPSSQ

Group M Virus (isolate ARV2/SF2)

MGARASVLSG GELDKWEKIR LRPGGKKKYK LKHIVWASRE LERFAVNPGL LETSEGCRQI
LGQLQPSLQT GSEELRSLYN TVATLYCVHQ RIDVKDTKEA LEKIEEEQNK SKKKAQQAAA
AAGTGNSSQV SQNYPIVQNL QGQMVHQAIS PRTLNAWVKV VEEKAFSPEV IPMFSALSEG
ATPQDLNTML NTVGGHQAAM QMLKETINEE AAEWDRVHPV HAGPIAPGQM REPRGSDIAG
TTSTLQEQIG WMTNNPPIPV GEIYKRWIIL GLNKIVRMYS PTSILDIRQG PKEPFRDYVD
RFYKTLRAEQ ASQDVKNWMT ETLLVQNANP DCKTILKALG PAATLEEMMT ACQGVGGPGH
KARVLAEAMS QVTNPANIMM QRGNFRNQRK TVKCFNCGKE GHIAKNCRAP RKKGCWRCGR
EGHQMKDCTE RQANFLGKIW PSYKGRPGNF LQSRPEPTAP PEESFRFGEE KTTPSQKQEP
IDKELYPLTS LRSLFGNDPS SQ

HIV-1 Gag Group M (isolate NY5)

MGARASVLSG GELDKWEKIR LRPGGKKQYK LKHIVWASRE LERFAVNPGL LETSEGCRQI
LGQLQPSLQT GSEELRSLYN TIAVLYCVHQ RIDVKDTKEA LDKIEEEQNK SKKKAQQAAA
DTGNNSQVSQ NYPIVQNLQG QMVHQAISPR TLNAWVKVVE EKAFSPEVIP MFSALSEGAT
PQDLNTMLNT VGGHQAAMQM LKETINEEAA EWDRLHPVHA GPIAPGQMRE PRGSDIAGTT
STLQEQIGWM THNPPIPVGE IYKRWIILGL NKIVRMYSPT SILDIRQGPK EPFRDYVDRF
YKTLRAEQAS QEVKNWMTET LLVQNANPDC KTILKALGPG ATLEEMMTAC QGVGGPGHKA
RVLAEAMSQV TNPATIMIQK GNFRNQRKTV KCFNCGKEGH IAKNCRAPRK KGCWKCGKEG
HQMKDCTERQ ANFLGKIWPS HKGRPGNFLQ SRPEPTAPPE ESFRFGEETT TPSQKQEPID
KELYPLASLR SLFGSDPSSQ

HIV-1 Gag Group M (isolate MN)

MGARASVLSG GELDRWEKIR LRPGGKKKYK LKHVVWASRE LERFAINPGL LETSEGCRQI
LGQLQPSLQT GSEERKSLYN TVATLYCVHQ KIKIKDTKEA LEKIEEEQNK SKKKAQQAAA
DTGNRGNSSQ VSQNYPIVQN IQGQMVHQAI SPRTLNAWVK VVEEKAFSPE VIPMFSALSE
GATPQDLNTM LNTVGGHQAA MQMLKETINE EAAEWDRLHP AHAGPIAPGQ MREPRGSDIA
GTTSTLQEQI GWMTNNPPIP VGEIYKRWII LGLNKIVRMY SPSSILDIRQ GPKEPFRDYV
DRFYKTLRAE QASQEVKNWM TETLLVQNAN PDCKTILKAL GPAATLEEMM TACQGVGGPG
HKARVLAEAM SQVTNSATIM MQRGNFRNQR KIIKCFNCGK EGHIAKNCRA PRKRGCWKCG
KEGHQMKDCT ERQANFLGKI WPSCKGRPGN FPQSRTEPTA PPEESFRFGE ETTTPYQKQE
KKQETIDKDL YPLASLKSLF GNDPLSQ
```

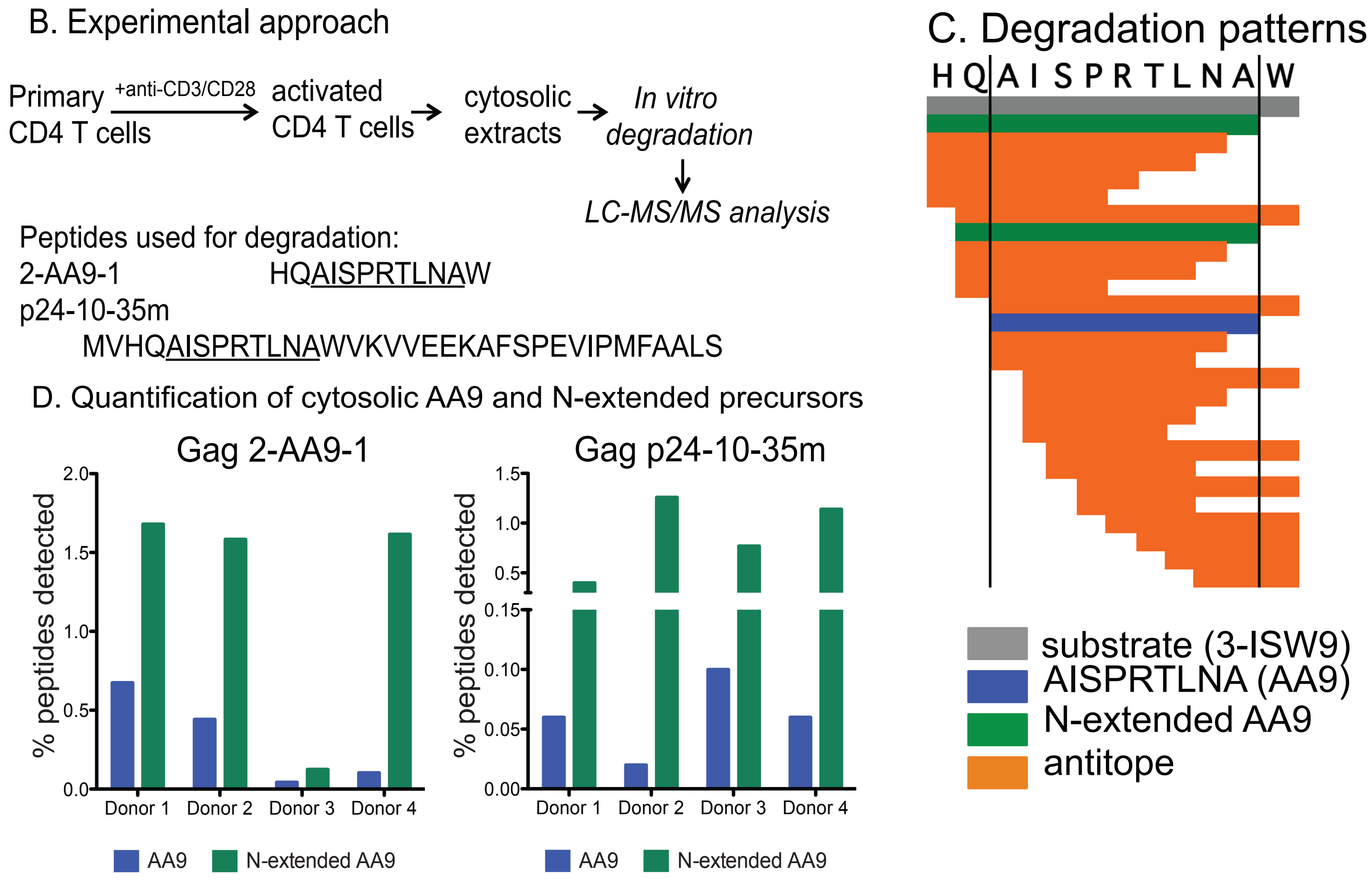

Supplement: S2 Fig — (A) Presence of AISPRTLNA peptide sequence (highlighted in yellow) within the proteome of various HIV-1 strains. (B) Experimental design of the degradation of long peptides in cytosolic extracts from activated CD4 T cells. (C) Peptides generated during the degradation of 2-AA9-1 include remaining substrate 2-AA9-1 (grey), the epitope AA9 (blue), N-extended precursors (green), antitopes (orange). (D) Relative quantity of AA9 (blue) and N-extended AA9 produced during a 2-hour degradation of 2-AA9-1 (left) or of p24-10-35m (right) in cytosolic extracts of activated CD4 T cells from four healthy donors. N-extended AA9 correspond to 1- and 2-aa extended for 2-AA9-1 and up 3-AA9 for the 35-mer. (PDF) [file ppat.1005421.s002.pdf]
